# 3D imaging of neuronal inclusions and protein aggregates in human neurodegeneration by multiscale x‐ray phase‐contrast tomography

**DOI:** 10.1111/bpa.70044

**Published:** 2025-10-01

**Authors:** Jonas Franz, Jakob Reichmann, Marina Eckermann, Thea Würfel, Artur Groh, Syed Fatima Qadri, Katja Schulz, Brit Mollenhauer, Christine Stadelmann, Tim Salditt

**Affiliations:** ^1^ Department of Neuropathology Göttingen University Medical Center Göttingen Lower Saxony Germany; ^2^ Aligning Science Across Parkinson's (ASAP) Collaborative Research Network Chevy Chase Maryland USA; ^3^ Göttingen Campus Institute for Dynamics of Biological Networks University of Göttingen Göttingen Lower Saxony Germany; ^4^ Max Planck Institute for Dynamics and Self‐Organization Göttingen Lower Saxony Germany; ^5^ Institute for X‐ray Physics University of Göttingen Göttingen Lower Saxony Germany; ^6^ ESRF, The European Synchrotron Radiation Facility Grenoble Auvergne‐Rhône‐Alpes France; ^7^ Institute of Applied Physics University of Bern Bern Switzerland; ^8^ Paracelsus‐Elena‐Klinik Kassel Hesse Germany; ^9^ Department of Neurology Göttingen University Medical Center Göttingen Lower Saxony Germany; ^10^ Cluster of Excellence “Multiscale Bioimaging: from Molecular Machines to Networks of Excitable Cells” (MBExC) University of Göttingen Göttingen Lower Saxony Germany; ^11^ Deutsches Zentrum für Neurodegenerative Erkrankungen (DZNE) Göttingen Lower Saxony Germany

**Keywords:** Lewy bodies, neurodegeneration, neuroimaging, protein aggregates, synchrotron radiation, x‐ray phase‐contrast tomography

## Abstract

This study leverages x‐ray phase‐contrast tomography (XPCT) for detailed analysis of neurodegenerative diseases, focusing on the three‐dimensional (3D) visualization and quantification of neuropathological features within fixed human postmortem tissue. XPCT with synchrotron radiation offers micrometer and even sub‐micron resolution, enabling us to examine intra‐ and extraneuronal aggregates and inclusions such as Lewy bodies (LBs), granulovacuolar degeneration (GvD), Hirano bodies (HBs), neurofibrillary tangles (NFTs), β‐amyloid plaques, and vascular amyloid deposits in three dimensions. In the reconstructions, we identified the highest electron densities in Hirano and LBs, while NFTs exhibited no significant increase in XPCT contrast. Using cutting‐edge high‐resolution x‐ray synchrotron beamlines, we were now able to detect even detect subcellular differences in electron densities found in GvD. Small‐scale inhomogeneities of the electron density were also detected in LBs, potentially relating to inclusions of organelles. Additionally, we reveal here a peculiar 3D geometry of HBs and demonstrate the co‐occurrence with GvD in the same neuron. These findings underscore the potential of XPCT as a powerful, label‐free tool for spatially resolved neuropathological investigations, opening new avenues for the systematic 3D characterization of inclusions and aggregates in neurodegeneration.

## Graphical abstract

x‐ray phase‐contrast tomography enabled three‐dimensional, label‐free visualization and quantification of protein aggregates in neurodegenerative diseases with isotropic submicrometer resolution. The study revealed distinct electron density patterns in Lewy bodies, Hirano bodies, granulovacuolar degeneration, and amyloid plaques, highlighting their subcellular structure and spatial organization.

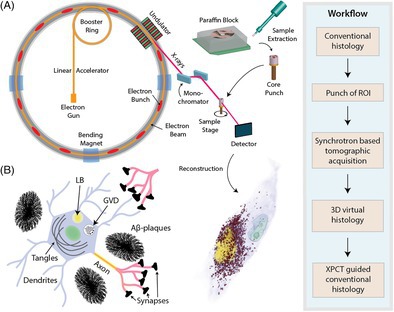

## 1 INTRODUCTION

Neurodegenerative diseases are characterized by specific cellular and extracellular protein aggregates. Studying their three‐dimensional (3D) and subcellular localization and their structural composition is crucial to gain further insights into the underlying pathological processes. Alzheimer's disease (AD) and Parkinson's disease (PD) are the most prevalent neurodegenerative diseases, and both are characterized by intraneuronal aggregates consisting of hyperphosphorylated tau in AD and α‐synuclein in PD. To date, the comprehensive visualization and quantitative assessment of these neuronal aggregates, alongside extracellular β‐amyloid plaques (β‐APs) or co‐occurring cerebral amyloid angiopathy (CAA) within a 3D histological context has remained technologically challenging. For AD and PD but also for rare diseases, for example, progressive supranuclear palsy, a description of stages and of spreading of protein aggregates across the brain is in use to diagnose and estimate the neuropathological involvement [1, 2, 3, 4, 5, 6, 7, 8, 9]. These staging systems have to take into account the complex and folded 3D architecture of the brain with its fiber connections but usually rely on sliced two‐dimensional planes. In addition, the frequent coexistence of neurodegenerative pathologies in approximately 50% of either AD or PD cases underscores the need for a method enabling a quantitative 3D assessment of pathological aggregates and inclusions at subcellular resolution with comparable quality to conventional histology [1, 10].

This gap between demand and capability of 3D histological imaging has recently been narrowed by x‐ray phase‐contrast tomography (XPCT). As a non‐destructive x‐ray technique it offers high penetration, scalable resolution, and sufficient contrast for unstained native, liquid‐ or paraffin‐embedded tissue [11, 12, 13]. Based on high spatial coherence of synchrotron radiation (SR) and even laboratory μ‐focus sources, XPCT exploits phase contrast arising from free space wave propagation, and applicability for the study of neurodegenerative diseases has been demonstrated both for animal models as well as for human tissue. In mouse AD models, for example, XPCT allowed the quantification of cellular aging [14] and the assessment of plaque morphology [15, 16, 17] in mouse cerebellum [18, 19]. Further, XPCT was used to track spinal cord injury of rats in 3D or to track neuronal loss, blood–brain barrier damage, and inflammatory cell infiltration in experimental autoimmune encephalomyelitis (EAE) [20, 21, 22]. Beyond animal models, investigation of biopsies of human nervous tissue by XPCT [23]—also denoted as virtual histology—was used in [24] to resolve sub‐μm structures in the cerebellum, with notable changes in the cytoarchitecture observed for multiple sclerosis [25]. In [26], correlative imaging of XPCT and conventional histology was used to investigate AD‐related pathologies of the hippocampal cornu ammonis 1 (CA1) region, further investigated in [27], where we found an unexpected chromatin compaction of granule cells of the dentate gyrus. On a larger scale, imaging of an entire liquid‐embedded human brain was recently demonstrated, with the possibility to zoom in at certain areas of interest with voxel sizes down to 1 μm [28].

In this work, we implemented a multiscale XPCT approach combining parallel and cone‐beam illumination with highly coherent third and fourth generation synchrotron beams to cover a wide range of scales and to achieve high‐quality reconstruction of human central nervous system (CNS) tissue, based on optimized optics, phase retrieval, and reconstruction. We started out with scanning of larger volumes of paraffin‐embedded tissue, fully compatible with conventional neuropathology workflows, to capture regions of interest identified, for example, by immunohistochemistry. High‐resolution zoom tomography was then used to study these areas in their native 3D context, focusing on intraneuronal (e.g., Lewy bodies [LBs], granulovacuolar degeneration [GvD], Hirano bodies [HBs], neurofibrillary tangles [NFTs]) and extraneuronal aggregates (β‐APs as well as vascular amyloid deposits). In a label‐free approach exploiting the contrast given by varying electron densities in human tissue, we identified hallmark features of neurodegenerative pathologies, enabling both qualitative and quantitative analyses. Further, having the 3D reconstructions obtained by non‐destructive XPCT at hand, we then carried out proof‐of‐concept correlative immunopathological investigations. Thereby, we were able to compare electron densities of hallmark pathologies using the unique contrast mechanism of XPCT based on electron density variations in tissue. Note that for biological tissues, the electron density is in good approximation proportional to mass density. Hence, the 3D reconstructions provide important constraints for modeling biomolecular packing in aggregates and inclusions.

## 2 RESULTS

**FIGURE 1:**
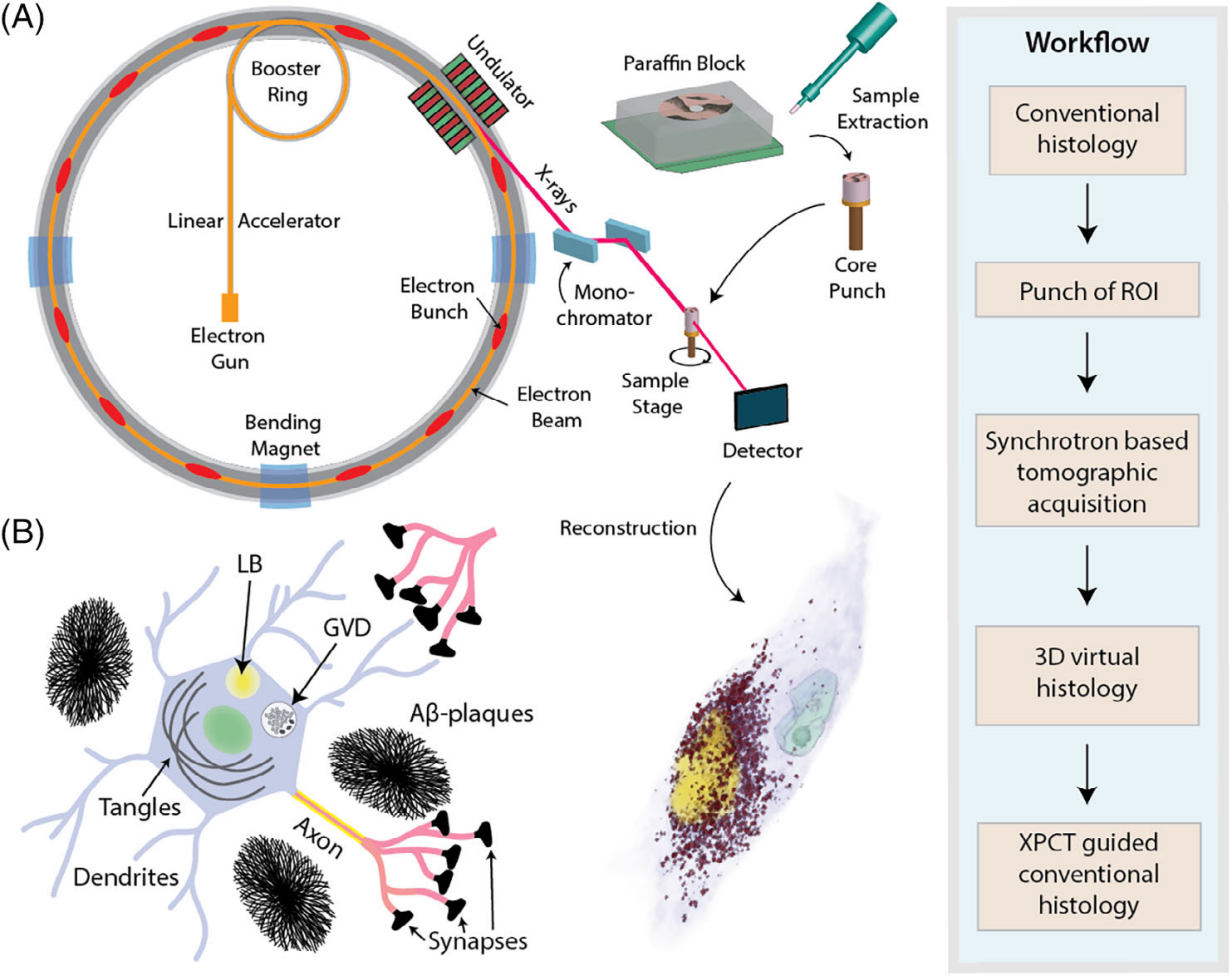
Experimental approach. (A) Extraction of small tissue punches from selected regions of interest of standard paraffin blocks. These punches were then subjected to x‐ray phase‐contrast tomography at two different beamlines. (B) Illustration of selected intra‐ and extracellular neurodegenerative pathologies and biomarkers. 3D, three‐dimensional; GvD, granulovacuolar degeneration; LB, Lewy bodies; XPCT, x‐ray phase‐contrast tomography.

Postmortem formalin‐fixed and paraffin‐embedded (FFPE) brain tissue from patients with neuropathologically confirmed AD (*n* = 3), PD (*n* = 1), and CAA (*n* = 1), as well as age‐matched controls (CTRL, *n* = 3), was selected for SR measurements. Regions of interest in the CA1 region of AD patients were identified by co‐immunohistochemistry for hyperphosphorylated tau and β‐amyloid. Temporal isocortex was assessed in CAA, whereas substantia nigra was examined in the PD patient tissue (for patient details, see Table 1). Full 3D tomograms of tissue punches of 1 mm diameter from the respective regions of interest (see Figure 1 for an overview of the experimental approach) were recorded using two different configurations: the SR1‐setup, a parallel‐beam configuration at DESY (PETRA III, Hamburg, Germany), for imaging overviews and calibration of electron density measurements, and the SR2‐setup, a cone‐beam configuration at ESRF (ID16A, Grenoble, France) for high‐resolution investigations (see Section 4 and Supporting Information S1 for detailed experimental setup, including Figures S1–S4, Tables S1 and S2).

**TABLE 1:** Patient characteristics.

Patient	Diagnosis	Age	Sex	Region of interest
# 1	AD (A3B3C3)	61–65	Male	CA1
# 2	AD (A3B3C3)	91–95	Male	CA1
# 3	PD	76–80	Male	SN
# 4	PART and CAA	86–90	Male	CTX, CA1
# 5	AD (A3B3C3)	81–85	Male	CA1
# 6	CTRL (A0B1C0)	51–55	Male	CA1
# 7	CTRL (A2B1C0)	96–100	Male	CA1
# 8	CTRL (A1B0C0)	65–70	Male	CA1

Abbreviations: AD, Alzheimer's disease; CAA, cerebral amyloid angiopathy; CA1, hippocampal cornu ammonis 1; CTX, cortex; PART, primary age‐related tauopathy; PD, Parkinson's disease; SN, substantia nigra.

### 2.1 Intraneuronal protein aggregates and vacuoles

#### 2.1.1 Lewy bodies in the substantia nigra closely associate with neuromelanin granules

**FIGURE 2:**
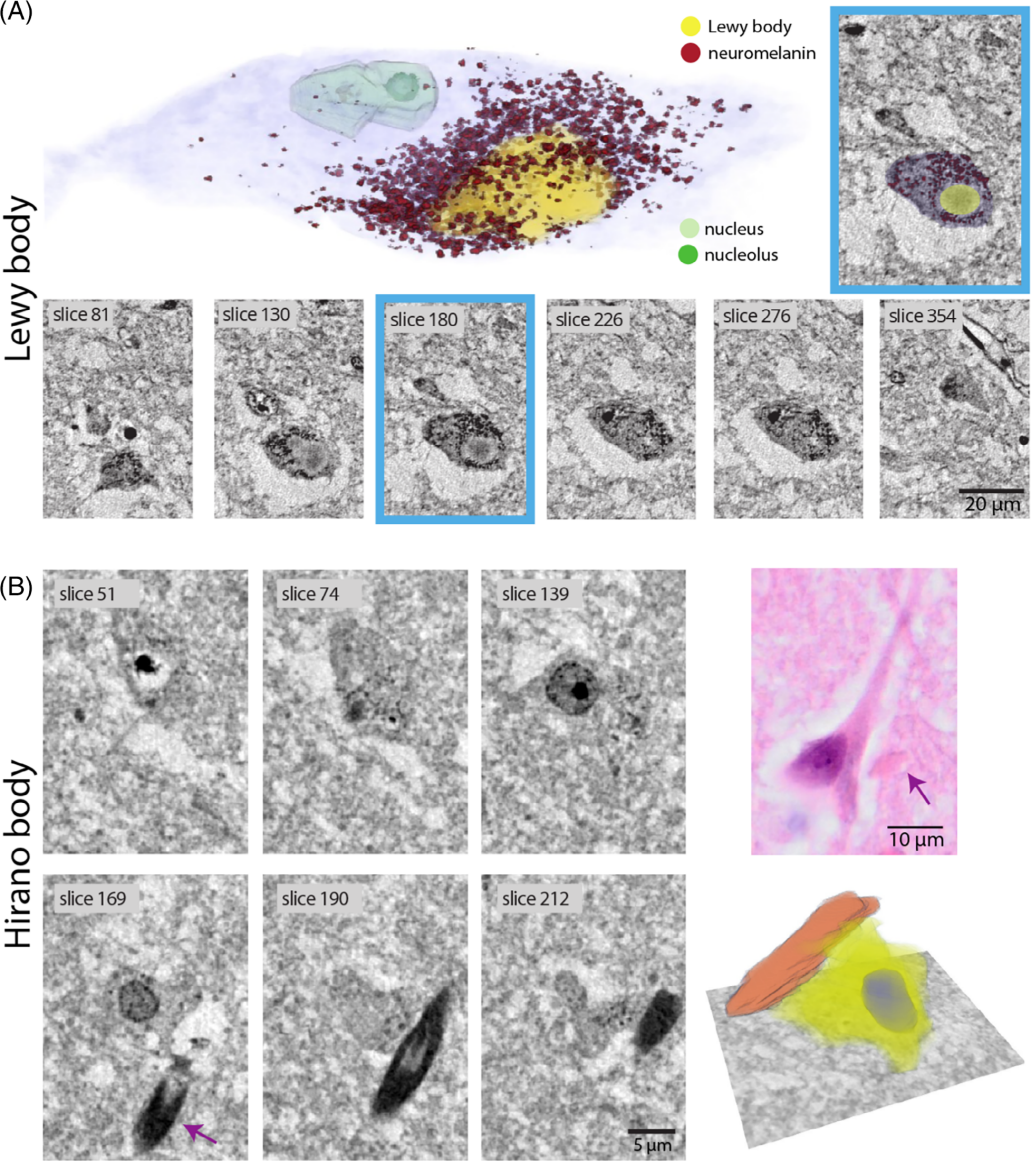
Lewy bodies (LBs) with neuromelanin granules and Hirano bodies (HBs) visualized in synchrotron micro‐beam computed tomography (μ‐CT) scans. (A) Virtual section through the reconstructed volume of a dopaminergic neuron (blue: neuron surface, yellow: LB, red: neuromelanin granules, light green: nucleus, dark green: nucleolus) recorded with the cone‐beam SR2‐setup and exemplary virtual serial slices of a LB. (B) Three‐dimensional reconstruction of a HB from the hippocampal cornu ammonis 1 region of an Alzheimer's disease patient with serial sections (SR2‐setup) and a HB in hematoxylin and eosin staining from the same tissue block.

LBs are the classical histomorphological hallmark of PD and show different morphologies depending on the neuronal subtype affected. Whereas LBs mainly consist of fibrillary α‐synuclein, other proteins as well as membranous organelles are embedded in the globular structure mostly presenting with a dense halo and a pale rim in classical histology [29, 30]. In the substantia nigra, locus coeruleus, dorsal nucleus nervi vagi, and other pigmented nuclei, LBs are in close spatial relationship with neuromelanin, which is absent in most other mammals and hypothesized to facilitate LB formation [31, 32, 33]. Neuromelanin was furthermore reported to play a pivotal role in neuroinflammatory processes during the progression of PD [34]. In the 3D reconstruction (Figures 2A and S8, Video S1), the layered substructure of the LB is easily discernible and reveals a dense homogeneous core with a less intense surrounding shell. Also, we found that LBs in pigmented neurons of the substantia nigra are embedded in the cluster of neuromelanin granules (NMGs) (see also α‐synuclein immunohistochemical [IHC] and SR1 measurements of XPCT punch in Figures S6–S9).

#### 2.1.2 3D arrangement of Hirano bodies and co‐occurrence with granulovacuolar degeneration

HBs are eosinophilic rod‐shaped structures that mostly occur in large pyramidal neurons of the subiculum and CA1 sector in patients with AD. Their origin and role in AD pathophysiology remain largely elusive, although their composition of actin filaments suggests a relation to cytoskeletal disruption. Light microscopic and electron microscopy (EM) studies suggest that HBs extend beyond the projected neuronal surface, presumably leading to a cell membrane protuberance. Figure 2B highlights the ability of XPCT to capture the 3D structure of HBs in human hippocampal tissue. Remarkably, HBs exhibit a pronounced contrast in XPCT compared to their typically rather inconspicuous appearance in hematoxylin and eosin (H&E) (Figure 2B, direct histological correlation see also Figures S12 and S13a). The representation in Figure 2B shows the 3D orientation of a HB (orange) with respect to the rest of the neuronal cell body (yellow) and the cellular nucleus (blue), demonstrating the lack of respect for the neuronal shape. This first human pathology‐based 3D representation demonstrates the relationship of the HB to the soma of the nerve cell but also shows its disruptive morphology beyond usual nerve cell borders. We also demonstrate here that HBs co‐occur with GvD in the same neuron (Figure 2B).

#### 2.1.3 Granulovacuolar degeneration fills large parts of the affected neuron

**FIGURE 3:**
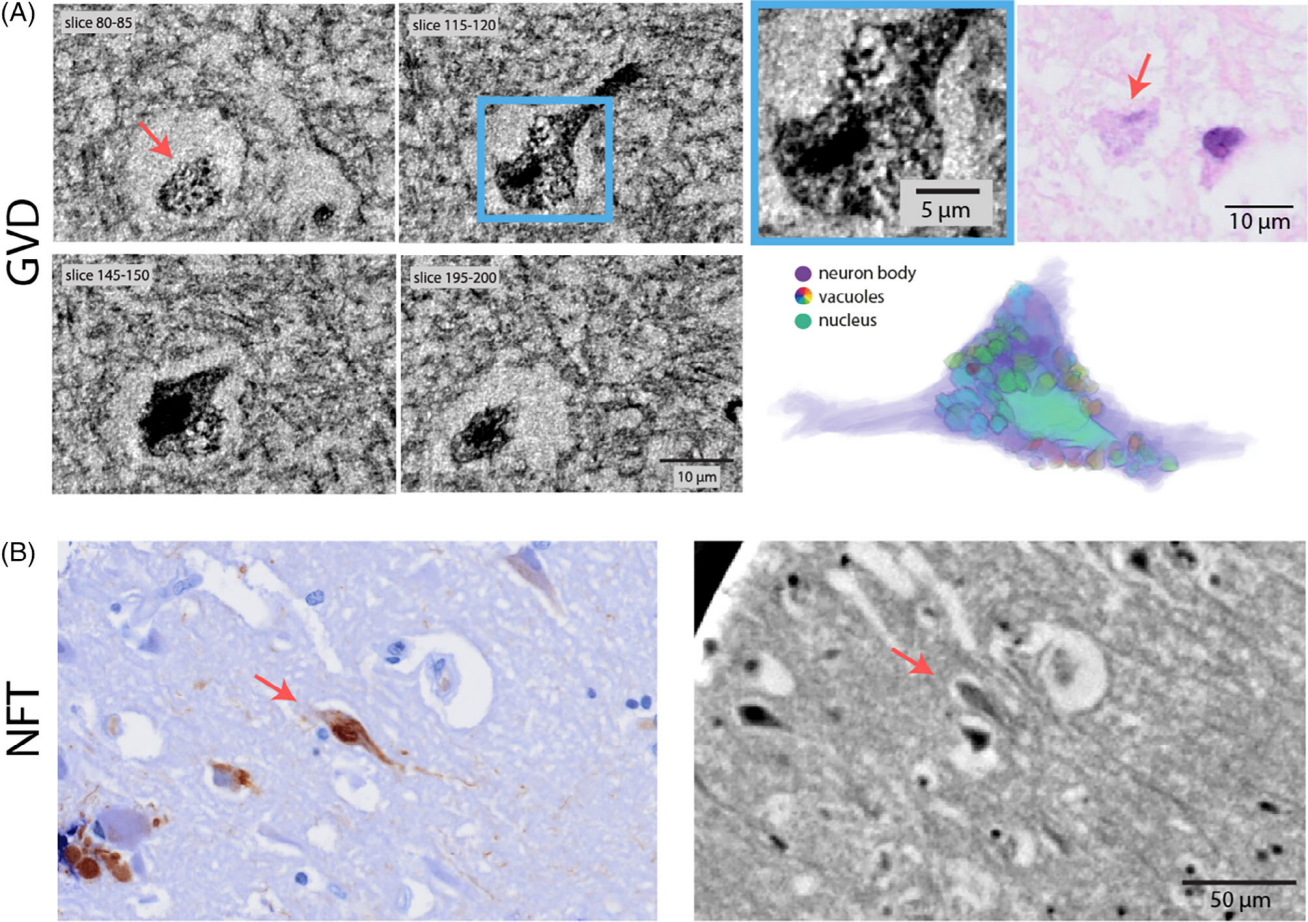
Granulovacuolar degeneration (GvD) and neurofibrillary tangles (NFTs) assessed by synchrotron micro‐beam computed tomography (μ‐CT) scans. (A) Three‐dimensional rendering of manually segmented granulo‐vacuoles from a hippocampal cornu ammonis 1 neuron in Alzheimer's disease with virtual serial sections of GvD (maximum intensity projections, SR2‐setup) and correlative histology (hematoxylin and eosin). (B) Tangle‐bearing neurons identified by correlative immunohistochemistry (AT8) and corresponding x‐ray phase‐contrast tomography scan (SR1‐setup).

GvD is a characteristic intraneuronal pathology predominantly occurring in the subiculum and CA1 region in patients with AD characterized by a grain‐like basophilic core surrounded by an optically empty vacuole. The presence in brain regions particularly vulnerable to AD pathology, its correlation with NFT density, and frequent occurrence in dysmorphic neurons suggest a close relationship to neurodegeneration. Also, GvD correlates with clinical dementia [7]. GvD has been proposed to reflect increased neuronal autophagy [35, 36]. Here, the 3D reconstruction enabled by XPCT allowed us to study how much of an affected neuron is occupied by GvD. Figure 3A shows an in‐part shrunken, but on the other hand bulging neuron from the CA1 hippocampal region of an AD patient overcrowded with GvD. The high resolution, contrast, and signal‐to‐noise ratio of the SR‐2 setup allow especially well for a segmentation of individual granules and their surrounding vacuoles (Figures 3A and S10). These annotations reveal a large assembly of vacuoles and granules. It appears as if vacuoles in part merge, which has recently been reported in an animal model of GvD using STED microscopy [37]. GvD seems to bulge the neuron asymmetrically.

#### 2.1.4 High‐resolution XPCT reveals similar contrast in neurons with or without neurofibrillary tangles

NFTs consist of hyperphosphorylated tau forming a helical and barely soluble structure. Neuropathological Braak staging of AD reflects an increasing presence of NFT‐bearing neurons from entorhinal to limbic to neocortical brain regions. Interestingly, the intracellular tau aggregates were difficult to identify in XPCT, in particular if compared to the easily discernible HBs or LBs. To ensure the proper identification of individual neurons with NFTs in XPCT, we optimized the correlative IHC analysis and applied a manual image registration approach using landmarks such as blood vessels (see Figures S5, S13, and S14). This technique allowed us to identify tangle‐bearing AT8‐positive neurons visible in both the IHC‐stained section as well as in XPCT, thus enabling, for example, comparative electron density measurements in tangle‐bearing and non‐tangle‐bearing neurons. Of note, only slight compositional heterogeneities of electron densities in the soma of affected neurons were observed. These features did not allow the detection of tangles by XPCT alone (Figure 3B).

### 2.2 Detection of β‐amyloid aggregation

Aside from intraneuronal aggregates, amyloid‐β species, which are cleaved extracellular peptides, have been central to neurodegenerative research for decades and are currently a molecular target for emerging therapies [38]. Though β‐APs and associated microglia activation can be measured using positron‐electron emission tomography, current clinical computed tomography (CT) scanners lack the capability to visualize different plaque types or to detect amyloid deposits in arterioles, veins, or capillaries in CAA. Our approach first involved a detailed characterization of β‐APs using the cone‐beam high‐resolution SR2‐setup. Secondly, the feasibility to investigate CAA with the parallel‐beam SR1‐setup was tested, which is important because larger tissue volume throughput can be achieved in this setting, which is required in order to search for these pathologies.

#### 2.2.1 Dense core of β‐amyloid plaques yields higher μ‐CT contrast

**FIGURE 4:**
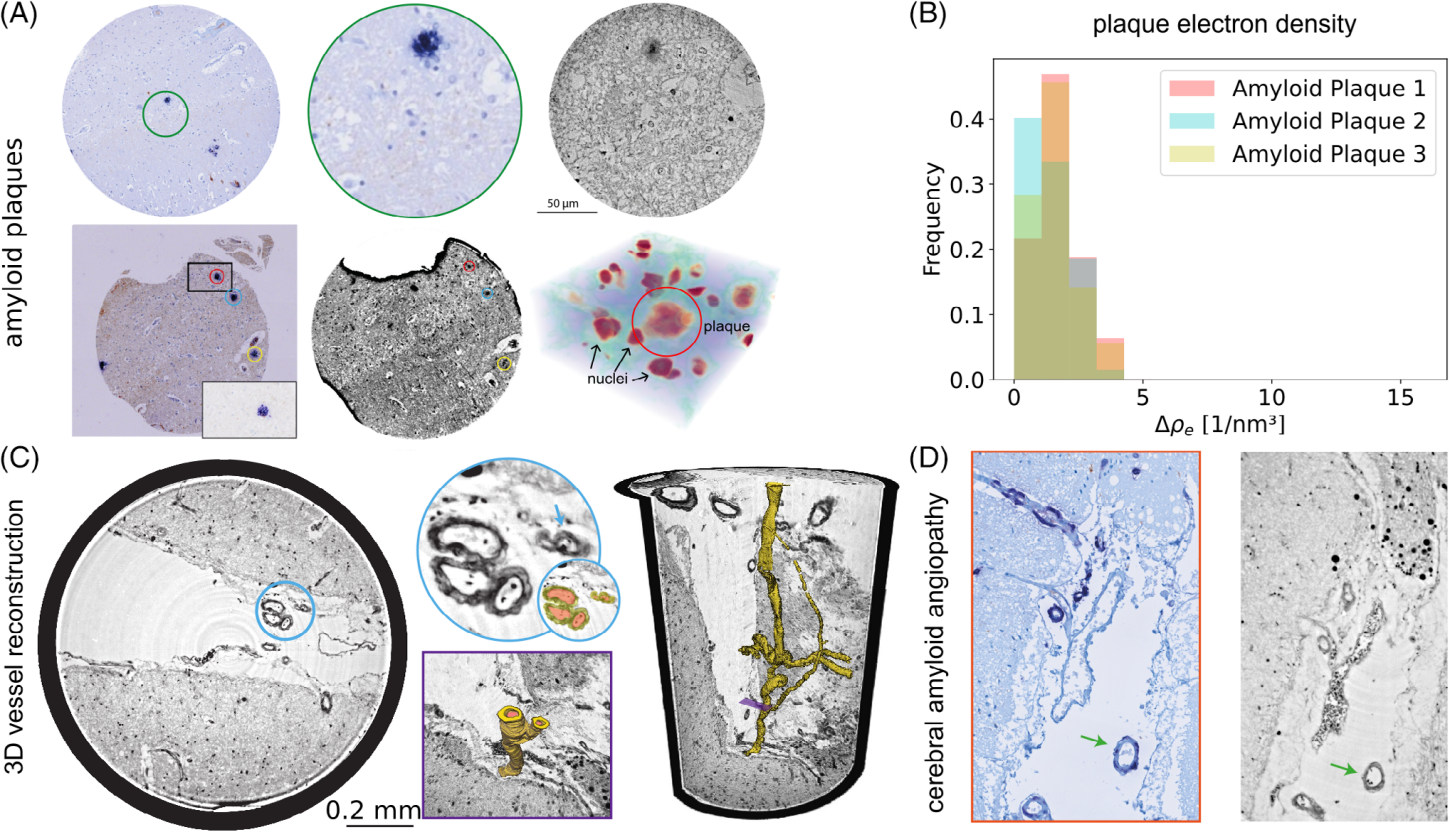
β‐Amyloid plaques (β‐APs), three‐dimensional (3D) vessel reconstruction, and cerebral amyloid angiopathy (CAA) visualized by x‐ray phase‐contrast tomography (XPCT). (A) Correlative immunohistochemical (IHC) of β‐APs (6E10, FastBlue) and high‐resolution XPCT (SR2‐setup). Second row: Low‐resolution XPCT with correlative IHC (6E10) and 3D representation of surrounding nuclei, blood vessels, and β‐AP (center, black arrow). (B) Electron density of three amyloid plaques identified by correlative immunohistochemistry. (C) Shown is a circular section through the 3D volume (lower right corner) with a magnified group of three blood vessels (blue) and a magnified part of the 3D rendered image (purple). (D) CAA with affected meningeal blood vessels recorded with the SR1‐setup. Correlative IHC (red) of the affected meningeal blood vessel (green arrow) confirmed vascular amyloid deposits.

β‐APs are heterogeneous extracellular protein deposits characterized by varying β‐amyloid/protein densities, different 3D structures, and distinct responses of the surrounding CNS microenvironment. Their presence is an indispensable feature for AD diagnosis. In our study, even the parallel beamline of the SR1 setup allowed us to delineate the comparatively dense protein core of β‐APs. Here, an effective pixel size of 650 nm enabled the detection and analysis of cored plaques in larger tissue volumes. The improved resolution offered by the SR2 setup with a cone‐beam even allowed it to identify the shell of a cored plaque with good contrast compared to the underlying glial matrix, as visualized in Figure 4A. Employing the correlation workflow outlined above, IHC images of stained β‐APs (using the 6E10 antibody) were juxtaposed to the corresponding XPCT measurements. Because of their size, the same amyloid plaque could easily be identified in both measurements. The second row of Figure 4A displays cored plaques measured in the SR1 beamline, clearly identified by correlative IHC, and their 3D distribution. The limitation of detecting the full plaque size, including the less compact shells, is also illustrated by the quantitative analysis of electron density in three histologically confirmed amyloid plaques. Consistent with their imaging behavior, the extracellular amyloid plaques seemed to be composed rather loosely, and only the core showed a slight increase in electron density (see Figure 4B and Section 2.3).

#### 2.2.2 Cerebral amyloid angiopathy

CAA may accompany AD, but may also occur independently. Characteristically, vessel wall structures are disrupted by β‐amyloid deposits, leading to vascular fragility and bleeding, and more rarely, vasculitis. XPCT is well known to allow for 3D blood vessel reconstruction, as demonstrated in Figure 4C, thus enabling a multiscale approach with larger volume throughput. Still, it is not clear if amyloid pathology in blood vessels leads to increased XPCT contrast. As for extracellular plaques, the amyloid deposits in CAA were only difficult to detect by XPCT. Applying correlative IHC on the exact same tissue section as measured by XPCT demonstrated only slightly increased contrast in affected vessel walls; see Figure 4D (also Figure S11).

### 2.3 Quantitative comparison of electron densities obtained by XPCT

Building on the unique contrast mechanism of XPCT to delve into hallmark features of human neurodegenerative diseases, the electron density of protein aggregates and GvD was assessed. Unlike conventional histology and IHC, which rely on staining‐specific amplification methods, the image formation of XPCT is directly linked to the electron density distribution. Using standardized acquisition and analysis, the electron density can be calculated from the phase shifts obtained by phase retrieval. Therefore, image gray values can be regarded as quantitative measurements of the local electron density. While this relationship can be more difficult to establish when absolute numbers are required, it is easier and more robust to provide analysis on local differences in electron density. Here, the paraffin embedding provides a well‐suited reference to “calibrate” electron density. As electron density (or correspondingly the mass density) effectively mirrors biological parameters such as fibril density or the presence of metal ions, it can provide important additional information on a given pathology. Of note, the standardized image acquisition process allows a direct comparison of neuronal and extracellular protein aggregates and structures, even across different diseases (for details on electron density calculation, see Supporting Information S1).

**FIGURE 5:**
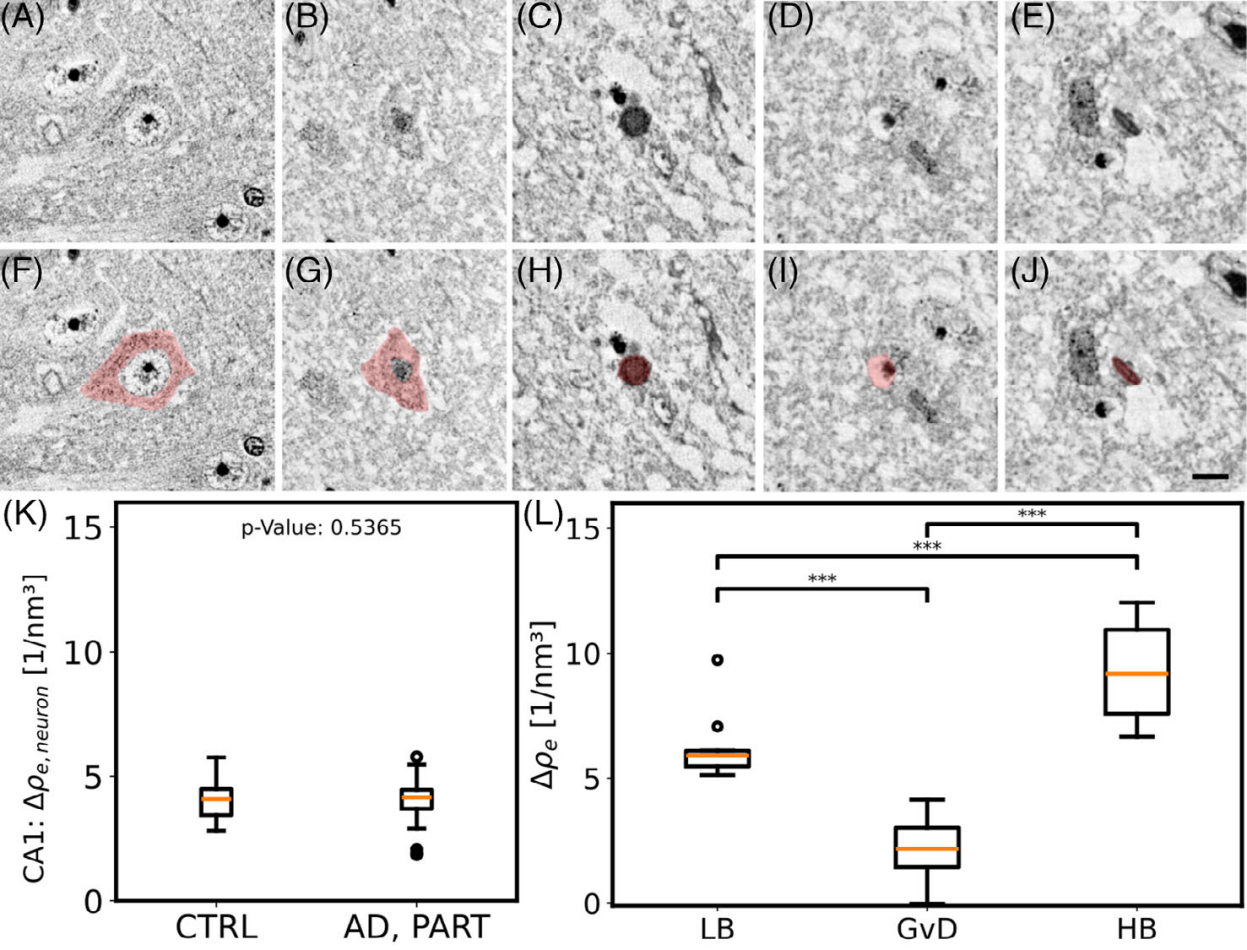
Electron densities of human intraneuronal neurodegenerative pathologies measured by synchrotron x‐ray phase‐contrast tomography scans. (A–J) showcases cross‐sectional calculated electron densities of neurodegenerative pathologies, with each column representing a different pathology, scale 5 μm. The second row (F–J) features the same section overlaid with a red region‐of‐interest delineating the electron density measurement for (K) and (L). Neuronal cytoplasmic electron density in (K) comparing tangle‐rich hippocampal cornu ammonis 1 (CA1) (B + G, *n* = 78, 4 cases) with tangle‐free control CA1 (A + F, *n* = 75, 3 cases) shows no significant difference (Mann–Whitney‐*U*‐test) with a mean elevation of ~4 electrons/nm^3^ compared to paraffin. Intraneuronal aggregates are presented in (L) with respective electron density for Lewy bodies (LBs) (C + H, *n* = 10, one case), granulovacuolar degeneration (GvD) (D + I, *n* = 18, two cases), and Hirano bodies (HBs) (E + J, *n* = 21, three cases) illustrating the distribution of electron densities within each pathological feature. ***Bonferroni corrected *p*‐value <0.001 of pairwise post hoc analysis of variance comparison by Mann–Whitney‐*U* test. AD, Alzheimer's disease; CTRL, age‐matched controls.

This study was powered to investigate tangle‐bearing neurons in AD (*n* = 3) and primary age‐related tauopathy (PART) (*n* = 1) compared to CTRL (*n* = 3) patients. The surprising lack of tangle contrast in XPCT did not allow for segmentation of tangle‐bearing neurons. Instead, neurons were manually segmented at their central soma while excluding HBs, the nucleus, and GvDs (~25 neurons per patient, *n* = 153 neurons in total). The electron density compared to paraffin exceeded about 5 e/nm^3^, but no significant difference between AD, PART, and CTRL was detected. Although this study might overlook small changes because of its limited *n*, we demonstrate here that the increase in electron density in tangle‐bearing neurons compared to normal neurons cannot be higher than ~2–4 e/nm^3^ (whisker range in Figure 5K). Using these samples, we are now able to provide comparisons to other aggregates.

Electron density assessment revealed that LBs (*n* = 10 LBs, 1 patient) show a higher and more compact electron density than tangles. We observed small inhomogeneities on the scale of 2–3 μm within the LB, predominantly in the so‐called “halo,” compatible with the notion that organelles may be encased in the fibrillary α‐synuclein network (see Figures 2A, 5C,H and figure 7 in [29]). Notably, the calculated quantitative electron density frequently, but not always, revealed a circular arrangement of these hypodense, potentially lysosomal organelles at the outer part of the LB, providing insights into the subcellular composition of this aggregate (see Figures 2A and S6–S9).

Using this approach, we could assign to HBs the highest electron densities in comparison to all other pathological aggregates and a comparable density to those NMGs (see Figure 5I,J,L), underscoring the utility of XPCT in differentiating between various intracellular aggregates based on their electron density profiles. GvD, in contrast, and as expected, did not display a homogeneous distribution of electron densities. However, we also could not ascertain a clearly bimodal distribution. This is suggestive of vacuoles showing a slowly increasing density toward their central “grain.”

In conclusion, electron density measurements by XPCT sort neurodegenerative aggregates from high‐density Hirano and LBs via low‐density β‐APs and NFTs to vacuoles from GvD.

## 3 DISCUSSION

Protein aggregates are a central hallmark of numerous neurodegenerative diseases instructing genetic and pathogenetic research; however, their formation, cytoplasmic embedding, and role for neuronal toxicity are not resolved. In this work, we leveraged XPCT of paraffin‐embedded autopsy tissue combined with correlative immunohistology to identify and compare disease‐defining pathological protein aggregates and inclusions in their full 3D and at subcellular scale. Our studies revealed a close spatial relationship of neurodegenerative protein aggregates to other intracellular structures, for example, neuromelanin, and distinct properties with regard to electron density. While we observed a particularly high electron density in HBs, NFTs were barely visible and required correlative imaging techniques. In GvD, instead of the expected bimodal density distribution, we found a gradient of increasing electron densities toward the central “grain,” suggesting increasing protein content. In summary, our work combines advanced x‐ray imaging techniques and correlative immunohistology in neurodegenerative diseases, demonstrating its capability for 3D assessment of protein aggregates and inclusions on the tissue and subcellular levels.

While the general compatibility of XPCT and FFPE samples has been previously established [26, 27, 39], we demonstrate here that improvements in resolution and contrast, facilitated by the specificities of the SR2 setup (see below), are sufficient to identify and to quantify also subcellular neuronal pathologies, such as LB, GvD, and HB. To our knowledge, some of these structures have never or only rarely been imaged in 3D before [40].

The size and orientation of protein aggregates relative to the cellular body, the nucleus, and other organelles may have significant implications, offering valuable insights into their development within the cell. For instance, the peculiar orientation of HB revealed through 3D virtual histology by XPCT (see Figure 2B) may give indications with regard to its mechanism of formation. Likewise, the noteworthy 3D association of neuromelanin around LB observed here (see Figure 2A) increases the evidence for an association of fibrillary α‐synuclein with neuromelanin pigments derived from extensive two‐dimensional histological studies [31]. The usual size of LB ranges from 5 to 25 μm in diameter, with a dense eosinophilic core of filamentous and granular material surrounded by radially oriented filaments [41, 42]. This core and the filamentous shell are likely to be reflected in the XPCT reconstruction in the form of elevated levels of electron density by XPCT. There is also recent evidence using confocal as well as super‐resolution stimulated emission depletion (STED) microscopy combined with EM and tomography that dense lysosomal structures and a shell of distorted mitochondria surround some of the LB inclusions (see figure 7 of [29]). In the present study, we were able to even identify these LB substructures, owing to the superior resolution provided by the SR2 setup.

While the 3D inspection alone allowed us to better understand the nature of the aggregates, their distribution in the tissue, and their relation to other intracellular structures, the quantitative analysis of XPCT contrast values revealed significant differences in electron densities between subcellular aggregates. By far, the highest electron density was found in HBs, which were found to correlate with relevant pathological aggregates like pTau, β‐amyloid, or α‐synuclein in a recent systematic study [43]. The high electron density is in line with previous EM studies using, for example, quick‐freeze deep‐etch technology, where HBs were demonstrated to consist of intracellular and densely packed fibrillar aggregates [44, 45, 46].

Compared to HBs the distribution of electron densities in LBs was lower, hinting toward less densely aggregated proteins or an inclusion of elements with higher electron density like metals in HB. The electron density in LBs was significantly higher than the mean of the GvD granules. However, GvD exhibited a broader distribution with a lower mean value accounting also for the vacuoles. The lowest values in GvD approximately reached a density of $\rho_{e}^{\mathrm{GvD}}\approx 0$ compared to paraffin (with $\rho_{e}^{\text{paraffin}}=328$). This would relate to the almost protein‐free vacuolar space in between granules.

Here, and to our knowledge for the first time, correlative immunohistochemistry was used to facilitate, at a single cell resolution, the identification of neurons with NFTs in XPCT. Even though the identification of tangle‐bearing neurons in the investigated samples was not yet possible by XPCT alone, the highly precise measurements and definitive identification of tangles through well‐established IHC allowed us to draw pertinent conclusions. Of note, and compared to HBs and LBs, tangles did not lead to a homogeneous increase in the electron density of affected neuronal cytoplasms. Relating this finding to existing EM and STED microscopy studies thus suggests that individual NFTs are surrounded resp. “diluted” by numerous subcellular organelles and do not fill up the neuron densely enough [47, 48, 49]. XPCT may therefore be complemented by highly advanced cryogenic electron microscopy resolving the atomic structure of protein aggregates, for example tau filaments in AD [50]. We can hypothesize from our studies that the pathologic effect of tangles may be rather related to a derangement of the cytoskeleton, especially the microtubules, obviously leading to various cellular dysfunctions, than to completely replacing existing subcellular organelles as observed in HBs. Our study also raises the question of whether extended XPCT methods implementing x‐ray contrast agents or increased resolution will be able to identify pathological tau filaments. Note that the resolution of XPCT has not yet reached its fundamental limits and can be expected to be further increased by ongoing instrumental improvements.

Also for β‐APs, correlative IHC further improved our detection sensitivity as demonstrated by high‐resolution images of the SR2‐setup (see Figure 4A). β‐APs were recently identified by XPCT in unstained human autopsy brain tissue by Chourrout et al. [15], reporting varying contrasts in mice and humans supposedly related to the degree of calcium and iron accumulation, in line with findings by Toepperwien et al. where mineralized plaques could be clearly observed even with in‐house micro‐beam computed tomography (μ‐CT) instrumentation or synchrotron x‐ray spectromicroscopy of cored plaques [24, 51]. Our electron density inspection of three β‐APs directly confirmed by histology revealed that the core of β‐APs showed a significantly higher electron density while the less dense shell was barely visible even in the high‐resolution SR2‐setup. One may wonder why β‐APs or NFTs are difficult to detect in XPCT. As for FFPE tissue, contrast is generated by the difference with respect to the embedding paraffin matrix, this can happen for organelles or structures which happen to exhibit similar density as the matrix. As a solution, contrast variation by different embedding media could be employed [11].

Also in the light of amyloid therapy‐related adverse effects, a better understanding of vascular amyloid deposition and microhemorrhage is relevant [52, 53]. CAA and β‐amyloid related angiitis (ABRA) represent a significant risk factors for intracerebral micro‐ and macrohemorrhages. Thus, a 3D visualization of microvascular changes may help to scrutinize the effects of amyloid depositions in blood vessels, also as an effect of amyloid targeting therapies. The highly precise μ‐CT strategy developed in this study (Figure 4B) demonstrates a new scale for the identification of even minor blood vessel changes invisible to angiography and for investigations of small vessel branching. While the voxel size and instrumental resolution (as determined for high contrast objects) are clearly sufficient to image the targeted pathologies at subcellular scale, the reconstruction of the FFPE tissue still exhibits substantial noise. In fact, as outlaid above, it is the low contrast of specific features such as unmineralized β‐APs which limit the 3D structural information gained rather than the instrumental resolution. For the SR2‐setup in particular, 50 nm (half period) resolution has been achieved in inorganic samples with high contrast or in metalized biological specimens [13, 54]. In order to reduce noise and to increase contrast in the present unlabeled FFPE tissues, one can either increase dose or decrease the photon energy E. As the phase shift Δϕ per resolution element scales with $E^{-1}$ (away from absorption edges), the contrast increases accordingly. Note that the diameter of the biopsy punches would clearly allow a reduction of E by at least a factor of two.

Future extension of this work may also include recently developed x‐ray stains [55, 56, 57, 58] and labels to improve contrast not only for distinct structural hallmarks of neurodegeneration covered in this work, but also for the individual cellular components, for example, axons, dendrites, or synapses, or the myelin sheath, which would open up a new perspective for x‐ray imaging of myelin‐associated diseases, such as multiple sclerosis.

In summary, our work combines XPCT and correlative immunohistology on paraffin‐embedded tissue specimens of neurodegenerative diseases. This approach allowed us to identify protein aggregates and other inclusion bodies and to reveal their 3D distribution in tissue, which is highly relevant, for example, for studies of the spreading of pathology. In addition, this multiscale approach enables subcellular 3D assessment of inclusion bodies, revealing insights into their relation to subcellular organelles. Upcoming technological developments will further increase the usefulness of this technology for the study of human CNS diseases.

## 4 METHODS

### 4.1 Sample preparation

Human brain tissue from individuals who underwent diagnostic autopsy in the context of routine clinical care was obtained from the archives of the Department of Neuropathology UMG in accordance with UMG ethics regulations. Small brain tissue blocks were dissected from 10% formalin‐fixed brain slices, dehydrated, and paraffin embedded (see [27]). One FFPE tissue block measured about 2 × 3 × 0.3 cm^3^. In total, nine samples from eight individuals were selected for the study, including seven samples from the hippocampal CA1 region (with immunohistochemically confirmed/excluded NFTs [see Section 4.2 and Figure S14]), one sample from the substantia nigra from a PD patient and one from the temporal cortex including the leptomeninges of a patient with CAA. Regions of interest for the punch biopsy for XPCT were defined on an adjacent histological section. To prepare the samples for XPCT image acquisition, cylindrical 1 mm biopsies were extracted from the paraffin blocks, inserted in a polyimide tube and placed on Huber pins. After imaging, these paraffin blocks were again embedded in paraffin and processed for direct comparison of further histological or IHC analysis.

### 4.2 Immunohistochemistry

Immunohistochemistry was performed on 2–3 μm thick paraffin sections. Pre‐treatment included hydrogen peroxide as well as formic acid (98%) (for Aβ immunohistochemistry only), blocking in 10% normal goat serum as well as heat antigen retrieval (citrate buffer, pH 6). Incubation of primary antibodies overnight (tau: mouse, clone AT8, Thermo Fisher Scientific, 1:100; β‐amyloid: mouse, clone 6E10, Zytomed Systems GmbH, 1:500) was followed by secondary antibody incubation either coupled to alkaline phosphatase (polyclonal goat anti‐mouse, Dako, 1:50) or to biotin (monoclonal sheep anti‐mouse, GE Healthcare Life Sciences, 1:100). Slides were developed using avidin‐peroxidase with diaminobenzidine (DAB) and/or Fast Blue.

### 4.3 Propagation‐based phase‐contrast imaging at the synchrotron

Synchrotron radiation allows for imaging with high coherence and brilliance and can cover multiple length scales down to sub‐100 nm resolution. In this work, we take advantage of a multiscale XPCT approach, using the parallel‐beam (SR1) configuration of the GINIX endstation installed at the P10 beamline of the PETRA III storage ring (DESY, Hamburg) [59] and the nano‐imaging beamline (SR2) ID16A (ESRF, Grenoble, France). While the former is optimized for larger field of view (FOV) with a parallel‐beam geometry, the latter is dedicated to propagation‐based holographic tomography of biological samples and operates in the hard x‐ray regime (17–33.6 keV). To achieve high resolutions, the beam is focused by a pair of Kirkpatrick‐Baez (KB) mirrors. Both provide quantitative phase contrast, which allows retrieval of information on the electron density in the sample, making them especially useful for biomedical applications. The two configurations can be used complementarily to study biological tissue at multiple scales. Details on the setup and acquisition parameters can be found in Supporting Information S1.

- SR1: The imaging procedure involved an overview scan using a FOV of approximately 1.5 mm. Therefore, a parallel‐beam configuration was employed, enabling continuous rotation and resulting in an overall scan time of approximately 2 min. The acquisition of single‐distance tomograms involved 3000 projections captured over a 360° rotation. To achieve a well‐defined photon energy for studying the tissue, a Si(111) channel‐cut monochromator was utilized, eliminating the broad band‐pass limitations inherent in in‐house CT systems. On the registration side, a high‐resolution detection system (Optique Peter, France) with a 50 mm‐thick LuAG:Ce scintillator and a 10× magnifying microscope objective [59] was coupled with the sCMOS camera pco.edge 5.5 (PCO, Germany). The camera performs with a maximum frame rate of 100 Hz, utilizing a rolling shutter and fast scan mode. This configuration yielded an effective pixel size of 0.65 μm, with an approximate total exposure time of 96 s. To achieve the desired imaging conditions, certain components such as KB‐mirrors, waveguide, and the fast shutter were removed from the beam path. Additionally, the beam size was adjusted to approximately 2 × 2 mm using an upstream slit system (refer to Figure S1).
- SR2: The nano‐imaging beamline ID16A provides a highly‐brilliant, low‐divergent beam optimal for 3D high‐resolution imaging of biological samples or other nanomaterials, for example, in batteries. With its multilayer monochromator and focusing KB‐mirrors, it allows for photon energies of either 17.1 or 33.6 keV and a photon flux of up to 4.1 × 10^11^ ph/s. The cone‐beam geometry enables a magnification of the projected pixels resulting in possible effective pixel sizes px_eff_ of less than 10 nm. The monochromaticity allows for a subsequent quantitative analysis and retrieval of sample characteristics such as electron density. Because of the strong magnification and high coherence, phase propagation can be very accurately recorded and reconstructed from the strongly holographic projections (F≪1). For detection, a XIMEA sCMOS based indirect imaging detector with 6144 × 6144 pixel (10 μm physical pixel size) and a 10× magnifying microscope objective was used. In this experiment the projections were binned (3 × 3), with effective pixel sizes ranging from 90 to 140 nm. Two thousand projections were recorded per scan with additional random sample displacement for every angle to correct for wavefront inhomogeneities and therefore avoiding ring artifacts [60]. Tomographic scans were acquired at four distances to account for zero crossings in the contrast transfer function (CTF) phase reconstruction [61]. From the four distances, the one with the highest resolution and largest FOV is combined, resulting in an “extended FOV” of 3216^2^ pixel in the resulting tomographic slices (refer to Figure S1).

### 4.4 Data processing

Projections were acquired and saved using the .tiff or .raw format. After flat field and dark image correction, operating in the holographic regime, phase retrieval was performed using either the CTF [61, 62], the nonlinear Tikhonov (NLT, [63]), or a Paganin‐based iterative scheme [64] (see Supporting Information S1). On the phase‐retrieved projections, ring removal techniques were conducted (only random displacement at SR2‐setup) as well as an automatic rotation axis correction. Tomographic reconstruction was then performed by either filtered back projection (parallel beam, SR1) or the Feldkamp‐Davis‐Kress (FDK, [65]) algorithm. Both techniques are implemented in the ASTRA‐Toolbox [66] for MATLAB and incorporated into the HolotomoToolbox [67]. Further details on the different reconstruction schemes can be found in Supporting Information S1.

### 4.5 Segmentation and visualization of cell components

After tomographic reconstruction of the recorded, phase‐retrieved projections and visual inspection, different structures were proposed for further analysis. After selection, segmentation was conducted mostly manually (via webKnossos interface [68]) or by using seeded watershed algorithms, deep learning‐based techniques (webKnossos [68], scalable minds GmbH, Potsdam, Germany) or simple thresholding. Subsequently, rendering software such as NVIDIA IndeX (NVIDIA, Santa Clara, USA), Avizo (Thermo Fisher Scientific, Waltham, USA), and ZEISS arivis (Carl Zeiss AG, Oberkochen, Germany) was used for a 3D visualization of the dataset. For additional post‐processing such as orthogonal views and maximum intensity projections, the Fiji software was used [69]. Details on segmentation and electron density calculations can be found in Supporting Information S1.

## AUTHOR CONTRIBUTIONS

**JF** and **JR** contributed equally to this work. **TS**, **CS**, **JF**, and **JR** conceived the experiments and analysis. **JF** selected and prepared the samples. **JR**, **TS** conducted the synchrotron experiments, together with **ME**, acting as local contact at the ID16A beamline. **KS**, **TW**, and **JF** performed correlative immunohistochemistry. **JR**, **AG**, and **JF** performed data processing, image reconstruction, and visualization. **JF**, **TW**, and **SFQ** annotated data and performed data processing. **JR**, **JF**, **TW**, and **CS** interpreted the results in terms of neuropathology. **BM** supported tissue procurement. **TS**, **CS**, **JF**, and **JR** wrote the manuscript. All authors reviewed the manuscript.

## FUNDING INFORMATION

The work was funded by the Deutsche Forschungsgemeinschaft (DFG, German Research Foundation)—Project‐ID 432680300—SFB 1456/A03 *Mathematics of Experiment* and under Germany's Excellence Strategy—EXC 2067/1‐390729940. This research was funded in part by Aligning Science Across Parkinson's ASAP‐020625 through the Michael J. Fox Foundation for Parkinson's Research (MJFF). Jonas Franz was supported by the UMG Clinician Scientist Program. Jakob Reichmann was supported by the Hertha Sponer College (MBExC).

## CONFLICT OF INTEREST STATEMENT

The authors declare no competing interests.

## ETHICS STATEMENT

Brain tissue blocks of patients with neurodegenerative diseases were obtained during diagnostic autopsy and retrieved from the archives of the Institute of Neuropathology, University Medical Center Göttingen, Germany. The tissue was used in an anonymized fashion. The study was approved by the ethics committee of the University Medical Center Göttingen (22/1/19).

## CONSENT

For the purpose of open access, the authors have applied a CC BY public copyright license to all author accepted manuscripts arising from this submission.

## Supporting information


**Data S1.** Supporting Information.

**Video S1.** Movie caption: Animated visualization of the 3D reconstruction and rendering of a dopaminergic neuron with a Lewy body, The data and sample relate to Figure 2 of the main MS, recorded with the cone‐beam SR2‐setup. Views of the electron density in parallel sections (gray value, animated by changing the planes) are combined with a rendering of the segmented Lewy body(blue: neuron surface, yellow: LB, red: neuromelanin granules, light green: nucleus, dark green: nucleolus). The rendering has been performed using the software Avizo (Thermo Fisher Scientific, Waltham, USA).

## Data Availability

Raw data were generated at ESRF and DESY. Raw data will be released and made public 2 years after the beamtime. All treated datasets, code, and protocols are publicly available as listed in the key resource table (https://doi.org/10.5281/zenodo.12566945).
