# Supplementary material for: 3D imaging of neuronal inclusions and protein aggregates in human neurodegeneration by multiscale x‐ray phase‐contrast tomography
Source: Brain Pathol. 2025 Oct 1;36(2):e70044. doi: 10.1111/bpa.70044 (PMC12861568; doi:10.1111/bpa.70044)
Supplement: Supplementary file 1 — Data S1. Supporting Information. [file BPA-36-e70044-s002.pdf]

3D imaging of protein aggregates in human neurodegeneration by multiscale X-ray phase-contrast tomography

Experimental Setup - X-ray phase-contrast tomography

In the following, all relevant experimental settings are tabulated. Hereinafter, the parallel beam setup at the GINIX endstation (Hamburg) is referred to as synchrotron radiation 1 (SR1), while the nano-imaging beamline at the ID16A at the ESRF (Grenoble) is referred to as SR2. Both setups are illustrated in Figure 1. Note that for SR2 the x_{01} distance refers to the first distance of a potential multi-distance tomographic scan. The dose deposited on the sample recording a single projection was estimated as:

$$D[Gy] = \frac{I_0 \tau E}{d \rho_m \mathrm{hv}}, \tag{1}$$

with the photon flux I_0, exposure time τ, photon energy E, attenuation length d, mass density ρ_m and the vertical and horizontal extent of the illuminated field of view in the sample plane (FOV), h and v, reciprocally. The photon flux on the sample was estimated as $\mathrm{I}_0 \approx 5 \cdot 10^{11}$ ph/s and $\mathrm{I}_0 \approx 4.1 \cdot 10^{11}$ ph/s for the parallel beam setup (SR1) and the high-resolution setup (SR2), respectively. The FOV was $1.6 \times 1.4\mathrm{mm}^2$ (h $\times$ v) for SR1, and $(0.21 - 0.45) \times (0.21 - 0.45)$ mm^2 (h $\times$ v) for SR2 (first distance). For the attenuation length d, $3 \cdot 10^{-3}$ m (SR1) and $0.59 \cdot 10^{-3}$ m (SR2) were used as computed by Henke et al. (1993) for a representative protein and tissue composite with stochiometry $C_{50}C_{30}N_9O_{10}S_1$. The mass density of paraffin embedded samples was estimated by 0.8 g/cm^3, as suggested in Kaye & Laby (1995).

Data Post-Processing

Phase Retrieval and Tomographic reconstruction

As a first step, all projections are flat- and dark field corrected, according to the scheme $\frac{\mathrm{proj}-\mathrm{dark}}{\mathrm{flat}-\mathrm{dark}}$. In propagation-based x-ray phase contrast imaging, a single projection on the detector is formed by free space propagation of the exit wave ψ_e behind the object, which in projection approximation is written as

$$\psi_e(y,z) = \psi_0(y,z)\ \tau(y,z)\ , \tag{2}$$

with ψ_0 the illumination function and $\tau(y,z)$ the complex-valued object transmission function, accounting for the attenuation and the phase shift introduced by the object.

Table 1: Experimental parameters for the parallel beam synchrotron setup SR1. FOV denotes the field of view (horizontal and vertical), x_{01} the source-to-sample distance, x_{12} the sample-to-detector distance, px_{eff} the effective pixel size, F the Fresnel number and D the dose.

	SR1
# projections	3000
FOV (h × v) (mm^2)	1.6 × 1.4
x_{01}	88 m
x_{12}	33 mm
px_{eff}	650 nm
Exp. time (s)	0.045
Tot. exp. time (s)	96
Energy (keV)	8.08
F	0.083
D (×10^4 Gy)	1.63

The intensity recorded in the detection plane then is given by as $I = |D_x(\psi_r)|^2|$, where D_x is the Fresnel propagator. As an approximative forward model in the holographic regime for weak objects, the contrast transfer function (CTF) in Fourier space is given by

$$\frac{\hat{I}_\omega(\mathbf{k}_\perp, x)}{I_0} = 2\pi\delta_D(\mathbf{k}_\perp) + 2\sin\frac{\lambda x(\mathbf{k}_\perp)^2}{4\pi}\hat{\phi}_\omega(\mathbf{k}_\perp) - 2\cos\frac{\lambda x(\mathbf{k}_\perp)^2}{4\pi}\hat{\xi}_\omega(\mathbf{k}_\perp) \ , \tag{3}$$

where $\mathbf{k}_\perp$ represents the components of the spatial frequency vector in the plane normal to the optical axis, λ the wavelength, while ϕ_ω and ξ_ω are the Fourier transformed attenuation and phase maps. Thus, we can observe an oscillatory contrast in phase and amplitude, both along the propagation axis x and in the $\mathbf{k}_\perp$plane. This implies zero crossing in the contrast transfer, resulting in missing frequencies and therefore loss of information. By recording tomograms at several different propagation distances, this can be compensated, thus filling up the reciprocal space and accounting for the missing frequencies.

To extract the encoded object information, phase retrieval algorithms are required, solving the inverse problem. For the SR1 experiments presented in this manuscript, CTF-based phase retrieval Cloetens et al. (1999) was carried out, assuming a slowly varying phase and a low-absorbing object (see CTF above), resulting in a regularized Fourier filter operation, which can be written for N different propagation distances as

$$\phi(\mathbf{r}_\perp) = \mathcal{F}_\perp^{-1}\frac{\sum_{m=1}^{N}[sin\chi_m + (\beta/\delta)cos\chi_m]\mathcal{F}_\perp[I_\omega^{exp}(\mathbf{r}_\perp, x) - 1]}{\sum_{m=1}^{N} 2[sin\chi_m + (\beta/\delta)cos\chi_m]^2 + \alpha(\mathbf{k}_\perp)}, \chi = \frac{\lambda z\mathbf{k}_\perp^2}{4\pi} \ , \tag{4}$$

Table 2: Experimental and reconstruction parameters for the high-resolution synchrotron setup SR2. # defoc. dist. denotes the number of recorded distances, FOV the field of view (horizontal and vertical), x_{01} the source-to-sample distance, x_{02} the source-to-detector distance, px_{eff} the effective pixel size, F the Fresnel number and D the dose. As for the reconstruction parameters, $lim1$ and $lim2$ denote the upper and lower cut-off frequency for the contrast transfer function and δ/β the ratio between refraction and attenuation in the sample.

	Lewy body	Hirano body	GVD	TE
# projections	2000	4000	4000	2000
# defoc. dist.	4	4	3	4
FOV (h × v) (mm^2)	0.45 × 0.45	0.29 × 0.29	0.21 × 0.21	0.45 × 0.45
x_{01} (mm)	53.1	34.7	36.5	53.1
x_{02} (m)	1.2	1.2	1.2	1.2
px_{eff} (nm)	140	90	90	140
Exp. time (s)	0.2	0.2	0.2	0.2
Energy (keV)	17.1	17.1	17.1	17.1
F (× 10^{-4})	5.4	2.2	2.2	5.4
lim1 (×10^{-3})	0.1	0.1	0.1	0.1
lim2	0.01	0.01	0.01	0.01
δ/β	80	80	80	80
D (×10^6 Gy)	4.2	20.1	38.4	4.2

with the frequency dependent regularization parameter $\alpha(\mathbf{k}_\perp)$ and $I_\omega^{exp}(\mathbf{r}_\perp, x)$ representing the experimentally measured intensity. The phase retrieval was carried out using the numerical implementation given in Lohse et al. (2020). For the SR2 data, an iterative phase retrieval scheme that extends Paganin's single distance method was used (Yu et al. (2018)). Following the phase retrieval of the projections and a ring-filter step as outlined in Münch et al. (2009), the tomographic reconstruction was carried out using either the filtered back-projection (FBP) method or the cone-beam algorithm (FDK), both of which were implemented in the ASTRA-toolbox (van Aarle et al. (2015)).

Segmentation and visualization of features of interest

After completing the tomographic reconstruction of the phase retrieved projections and subsequent visual inspection, a range of structures were put forth for further analysis. Depending on the particular dataset, different segmentation methods were explored. For structures with well-defined edges, seeded watershed algorithms implemented in the

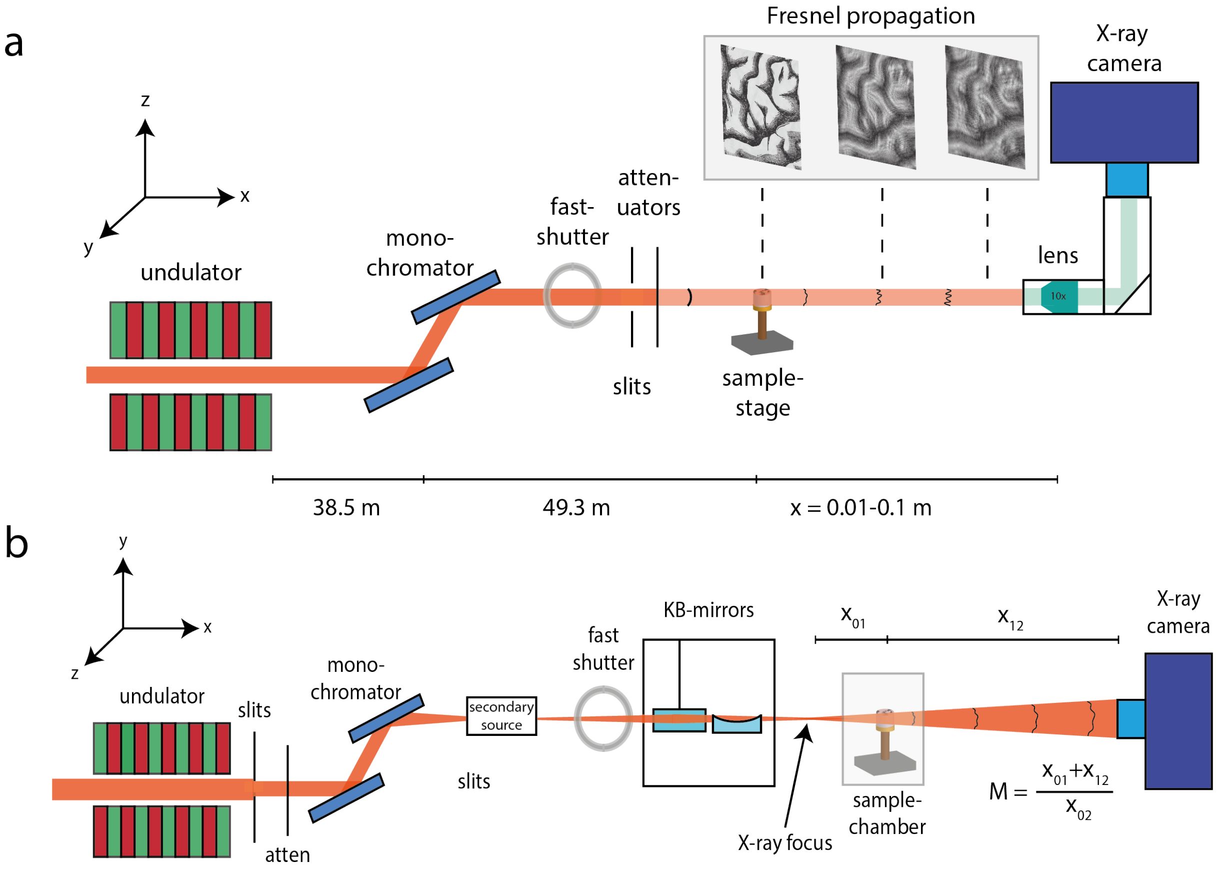

Figure 1: **a** Parallel beam setup at the GINIX instrument, P10 beamline, DESY, Hamburg (photo in supplementary document, detailed description in the text). **b** High-resolution holo-tomography setup at the ID16A beamline, ESRF, Grenoble (photo in supplementary document, detailed description in the text).

.

Carving option in the interactive learning and segmentation platform Ilastik (Berg et al. (2019)) was used (see Fig. 4 in main manuscript). If the contrast was sufficient, pixel classification was based on machine learning algorithms (also implemented in Ilastik), as an alternative to manual segmentation or simple thresholding which often encounters difficulties. For shape-based segmentation, machine learning and deep learning based tools implemented in the ZEISS arivis software (Carl Zeiss AG, Oberkochen, Germany) and webKnossos Boergens et al. (2017) (scalable minds GmbH, Potsdam, Germany) were applied. To this end, regions of interest (ROI) were manually annotated in a few slices, whereupon the features in the intermediate slices were automatically recognized and segmented by the software. To appropriately visualize the three-dimensional data, NVIDIA IndeX (NVIDIA, Santa Clara, US) and Avizo (Thermo Fisher Scientific, Waltham, US) was used, as previously described in Reichmann et al. (2023).

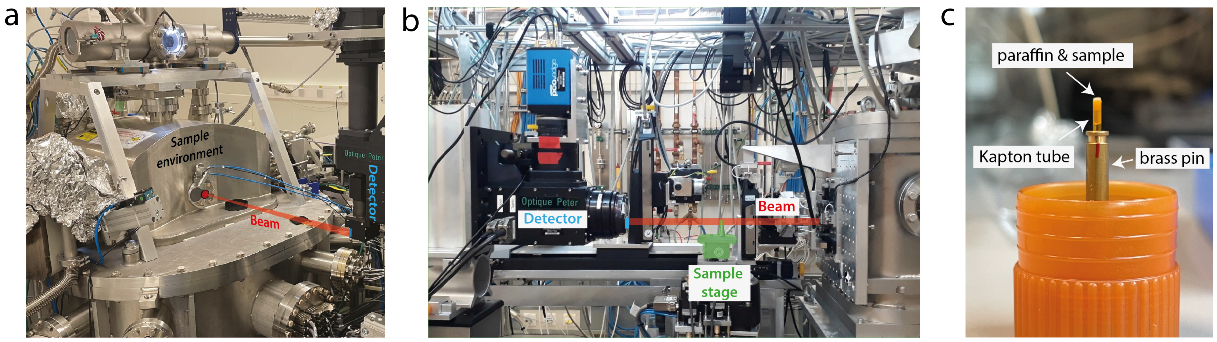

Figure 2: **a** Photograph depicting the SR2 setup at the ID16A beamline (ESRF, Grenoble, France). The sample is placed by a manually controlled gripper inside the sample environment in vacuum. After the exiting window the beam propagates through air before it hits the detector. **b** Photograph depicting the SR1 parallel beam setup at the P10 beamline (PETRA III, Hamburg, Germany). The monochromatic beam is attenuated by polished single-crystal attenuators (Si) and thereafter directly passes through the sample without any intermediate optical elements. **c** Sample as prepared for the experiment, biopsy surrounded by a Kapton foil inside Huber (brass) pin.

.

Electron Density Calculations

Since electron density calculations require a reference, empty spaces in the tissue blocks like ventricular space, enlarged perivascular ridges or larger erythrocyte and serum-free areas were annotated as a paraffin reference (according to the regions typically selected for white balance in standard light microscopy). Three ROIs per sample were annotated for the determination of the paraffin reference. An isotropic voxel size for the SR1-setup was given as $d_{Voxel} = 650$ nm. After phase retrieval and reconstruction, the electron density was calculated from the intensity of the reconstruction output $I(\vec{x})$ assigned to a position $\vec{x}$ in 3D, according to Robisch et al. (2020), Henke et al. (1993):

$$\Delta\rho_e(\vec{x}) = \frac{2\pi}{d_{Voxel}} \frac{\Delta\left(\bar{I}_{paraffin}, I_{tissue}(\vec{x})\right)}{r_0\lambda}. \tag{5}$$

$\Delta\rho_e$ is the electron density shift compared to paraffin and r_0 is the classical electron radius ($\approx 2.818 \cdot 10^{-6}$ nm).

For scans obtained at the SR2-setup each recording was aligned to electron density measurements from the same sample using the SR1-setup. To this end uniform structures like paraffin holes or blood clots were selected and identified in both recordings. SR2-setup paraffin normalized intensity values were mapped onto SR1-values using linear regression (see Fig. 3. This allowed electron density estimation also for subcellular structures like GvD identified in the SR2-setup only.

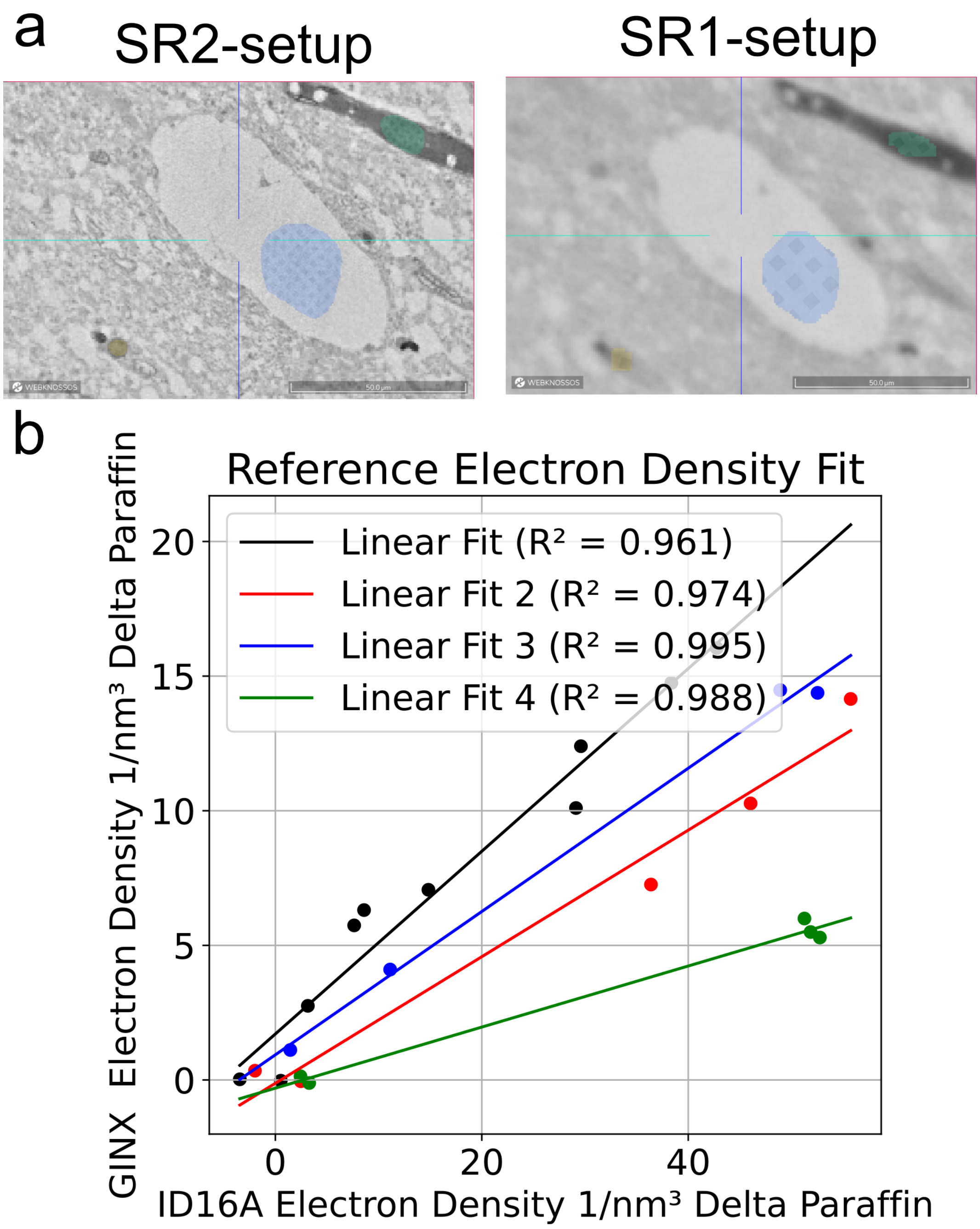

Figure 3: Linear regression to map SR2-pseudo electron density values on SR1-setup electron densities.

Estimation of spatial resolution

For an estimate of the spatial resolution, Fourier-shell-correlation (FSC) van Heel & Schatz (2005) was performed. To this end, a scan with 4000 acquired projections was divided into two equally sized datasets of 2000 projections each (even/odd) and separately reconstructed. FSC was then evaluated as a function of the spatial frequency (in units of $\frac{2\pi}{px}$ on a region-of-interest (1000^3 pixel), selected from the center of the volume as implemented in Lohse et al. (2020). The half-bit threshold was used as the criterion for the spatial resolution estimation with the according threshold curve plotted in Figure 4**b** alongside the FSC-curve. The intercept, which determines the maximum resolvable spatial frequency, was found at 0.8 $\frac{2\pi}{px}$, yielding a half-bit resolution of 355 nm. Since the dataset was split, this value is a conservative estimate.

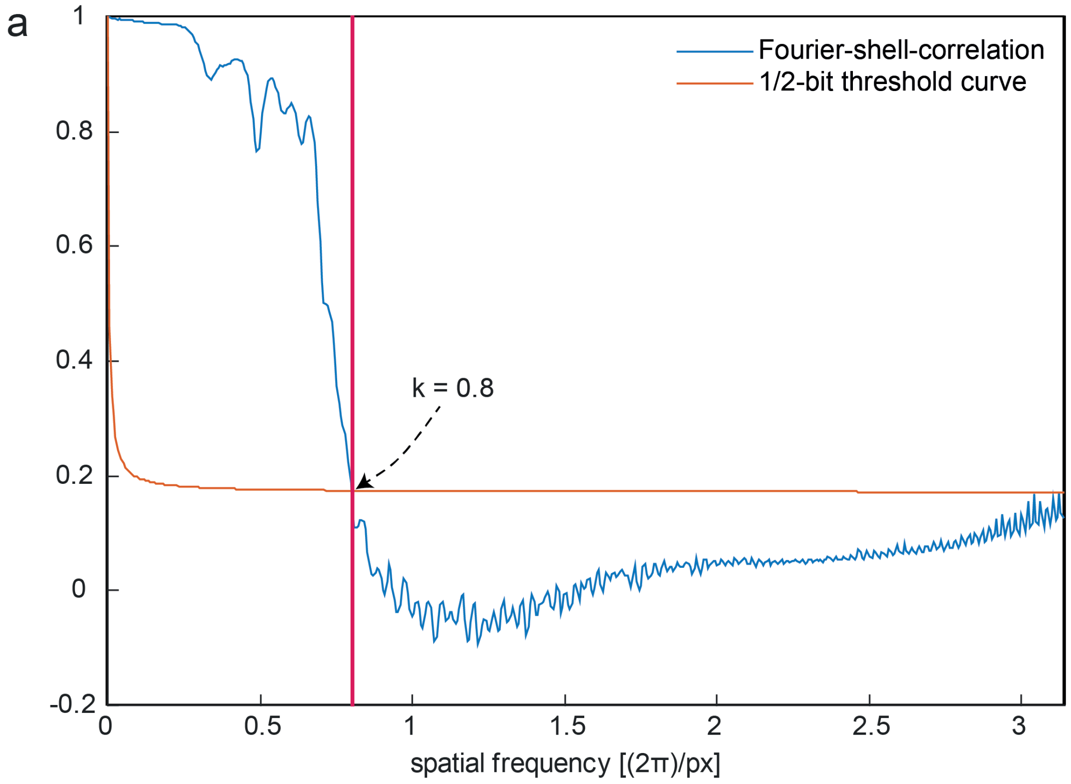

Figure 4: **a** FSC-curve plotted alongside half-bit threshold curve for a high resolution dataset acquired with the SR2-setup (Hirano-body dataset). Intercept at k =0.8 $\frac{2\pi}{px}$ yields a half-bit resolution of 355 nm.

Correlation of XPCT recorded tomographic reconstruction to histological sections

The procedure of a correlative comparison between different modalities, in this case stained histological slices investigated under a light microscope and slices from reconstructed XPCT-recorded volumes, is exemplified on a sample with an expected considerable amount of $A\beta$-plaques. After data acquisition by XPCT, the remaining 1

mm diameter paraffin biopsy was embedded back into an entire paraffin block. With guidance by the reconstructed volume, as common practice in histopathology, the tissue is cut with a microtome in 3 μm slices. These were then stained (H&E (Figure 5**a**, 6E10 (Figure 5**b**) or other antibody stains) and the slice is digitalized. In a next step, the XPCT volume was browsed until certain features were spotted, visible in the stained histological section. In this example, an elongated region was discovered where the sample matched almost perfectly (Figure 5**d,e**). Finally, the plane in the XPCT is rotated around the elongated axis previously found, until the other features matched. Since the histological section is not isotropic and has a thickness of several μm, finding a section where all features perfectly match is strictly speaking impossible. While in Figure 5**c** morphological features at the bottom and to the left part align well, this is not the case for the upper right part of the section.

By further tilting the slice, the plaques finally come into the FOV (Figure 5**f**).

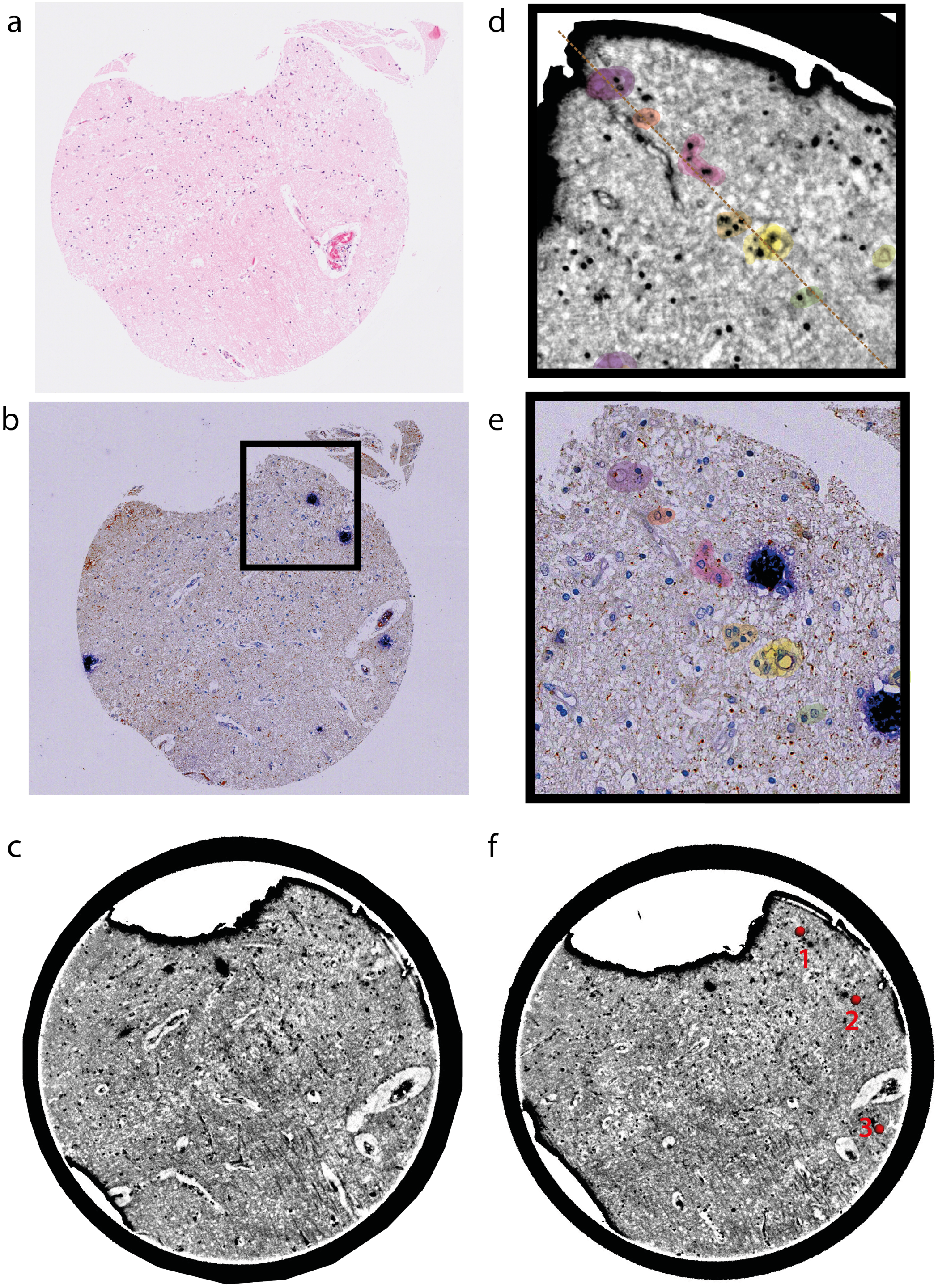

Figure 5: H&E stained (**a**) and 6E10 stained (**b**) slice from human hippocampal tissue (CA1) with a zoom into a plaque-rich area (**e**) and correlative slice in the XPCT dataset (**d**) recorded at the LS1 setup. (**c**) shows slice through 3D volume, where most features are aligned with the histological slice, while with further tilting the plaques become visible (**f**).

Supporting Figures: Further examples of Lewy bodies in 3D, of the SR1-setup, and adjacent immunochemistry

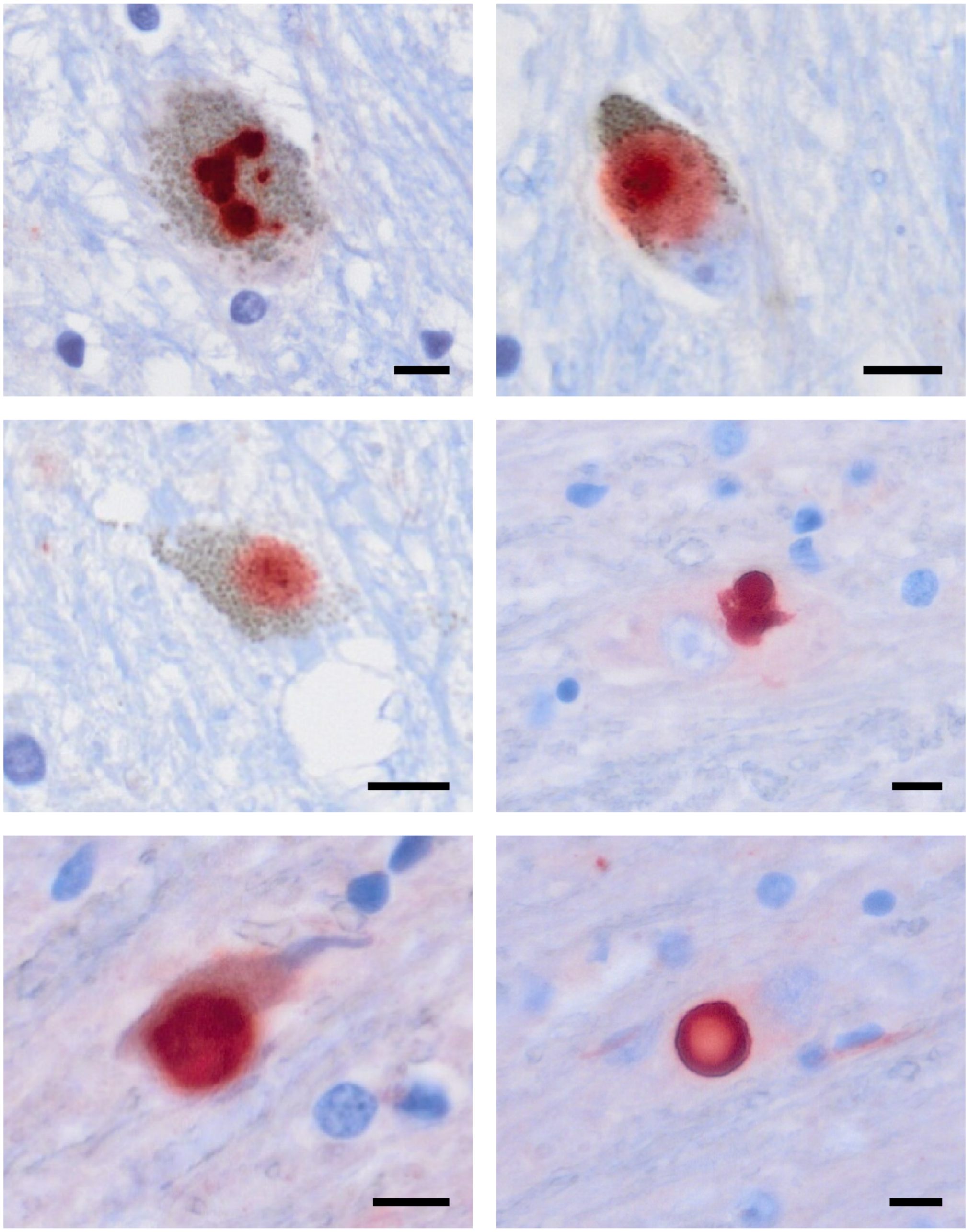

Figure 6: Exemplary Lewy bodies from the 1 mm punch stained for α-synuclein (LB509) after XPCT recording. Some Lewy body containing neurons show typical neuromelanin pigments while some neurons appear not pigmented in this section. Scale bar 10 µm

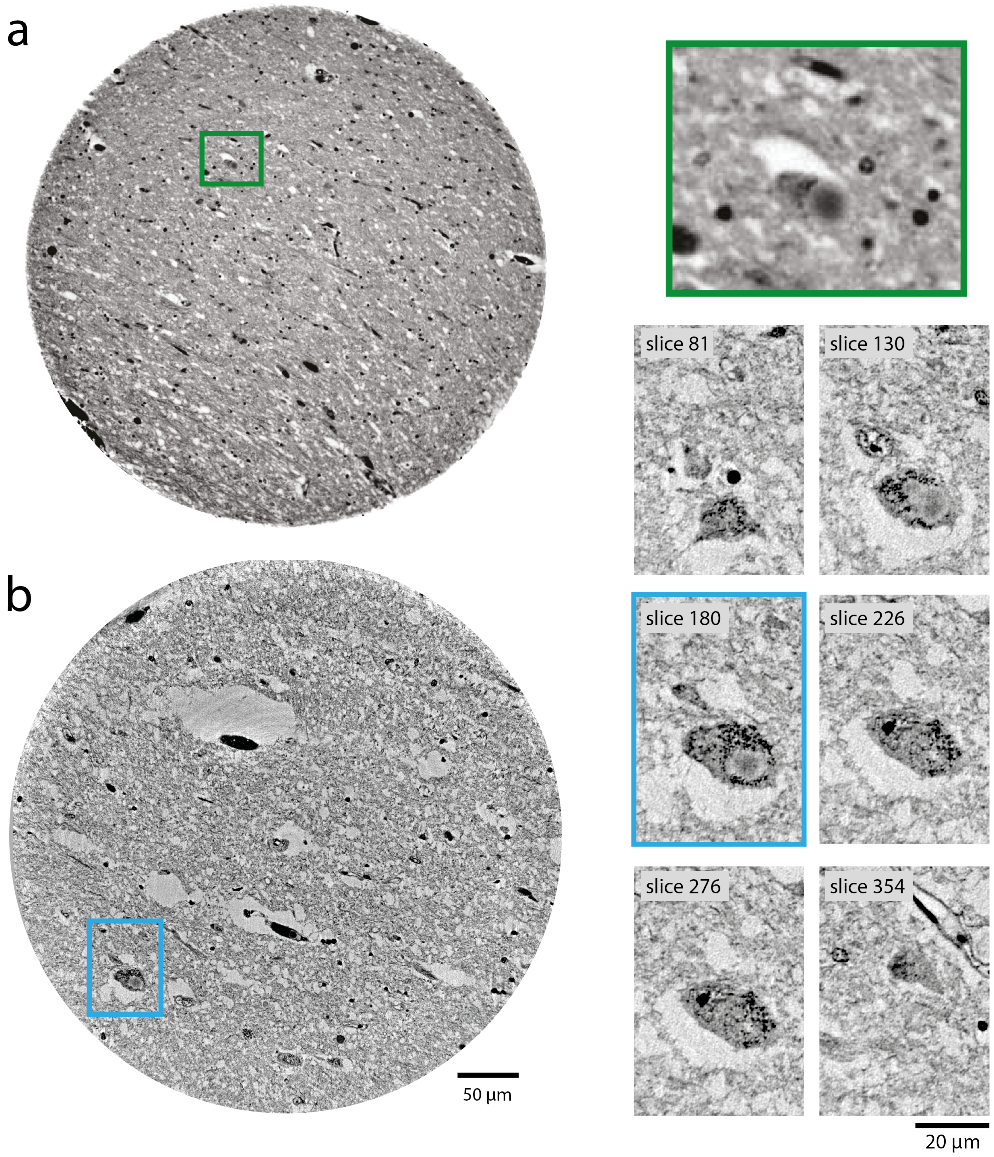

Figure 7: Comparison of SR1-setup and SR2-setup for Lewy body visualization. **a** Virtual section through the reconstructed volume of substantia nigra, recorded with the parallel beam setup (SR1, left), with a zoomed depiction of a Lewy body (right). **b** Reconstruction of the same sample from a high resolution scan recorded with the cone beam setup (SR2, left), and corresponding virtual sections through a Lewy body (right).

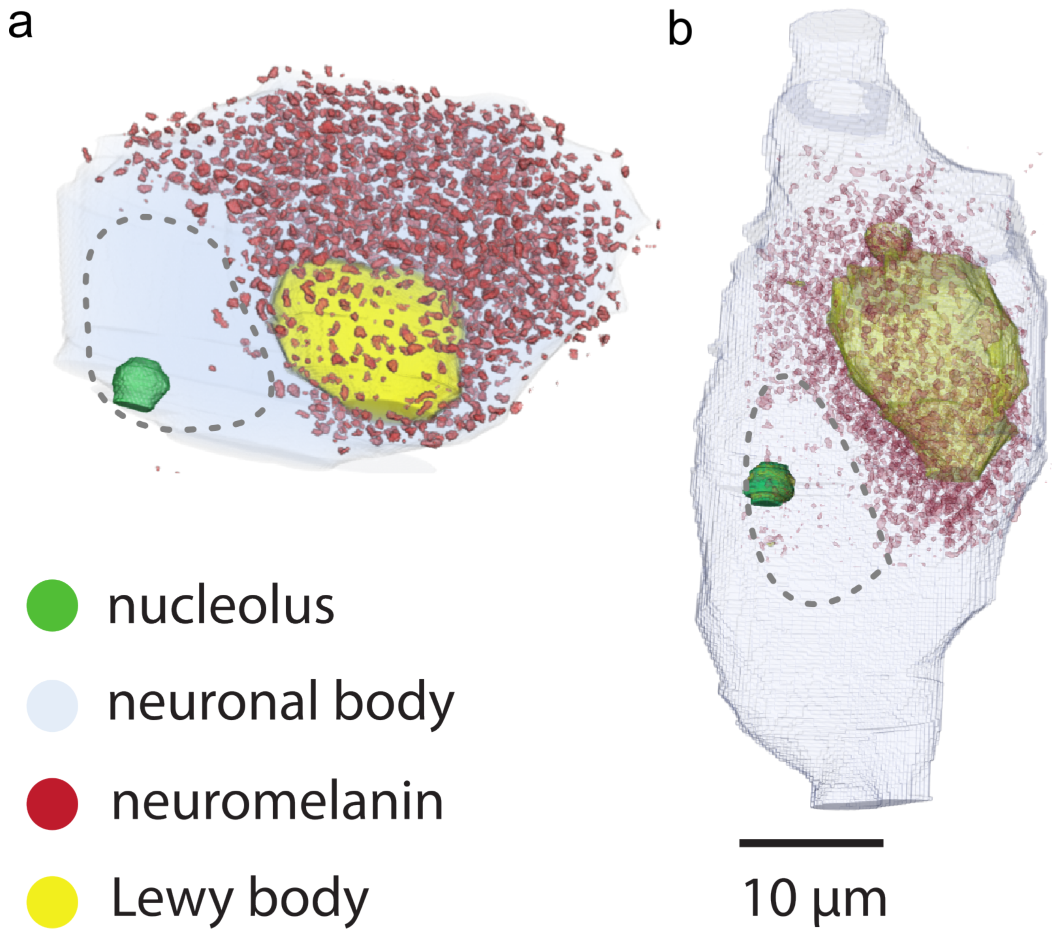

Figure 8: Further examples of Lewy bodies in the 3D context of pigmented neurons.

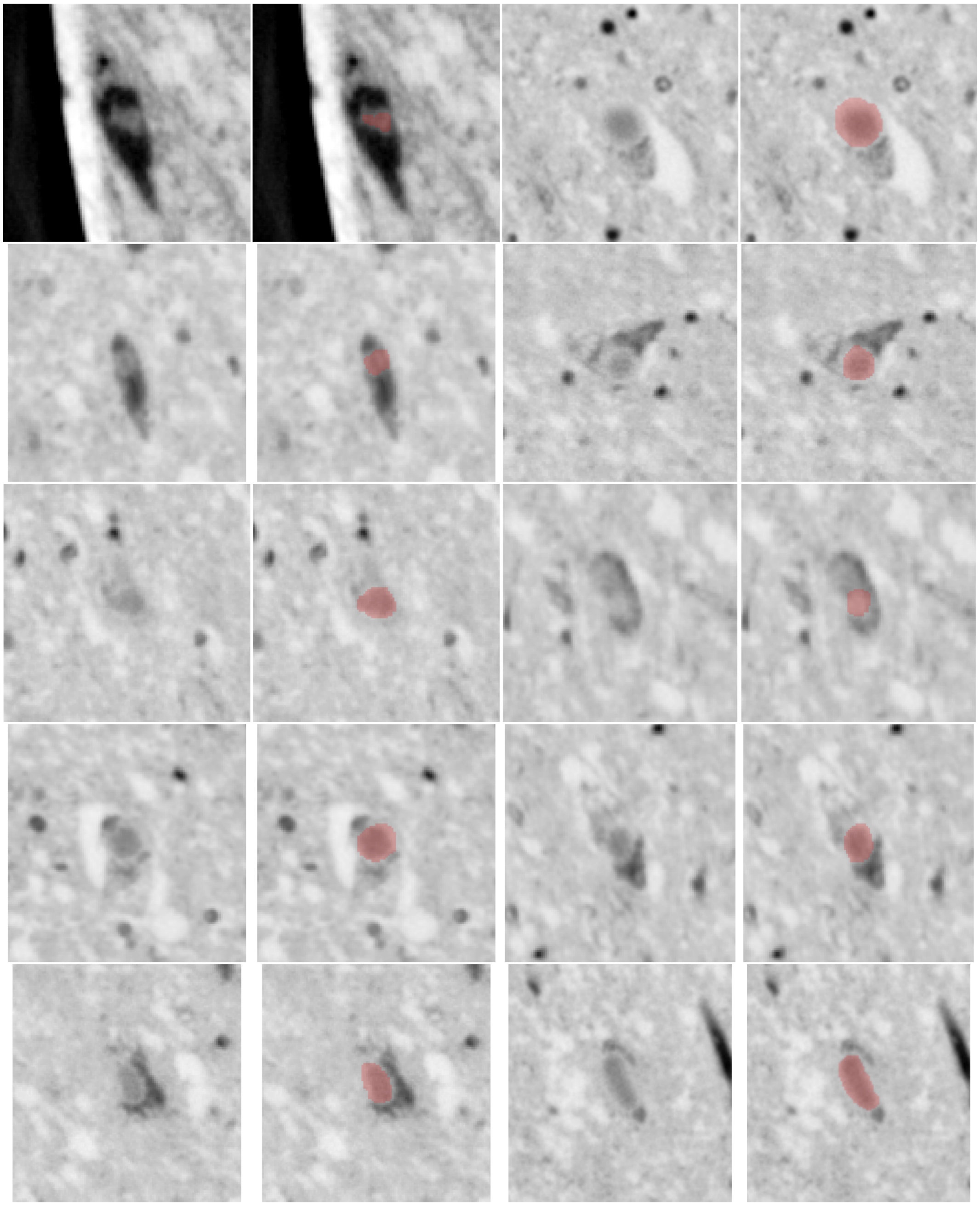

Figure 9: Several Lewy bodies identified at the SR1-setup with a red annotation mask. Please note that the 3D information available was used for the annotation to securely identify the LB; and that not all information could always be presented in a representative 2D slice. Note that neuromelanin granules are not clearly discernible at the SR1-setup and with this resolution appear like a homogenous dark area.

Supporting Figures: Correlation between high-resolution dataset recorded at SR2-setup and overview scan recorded at SR1-setup

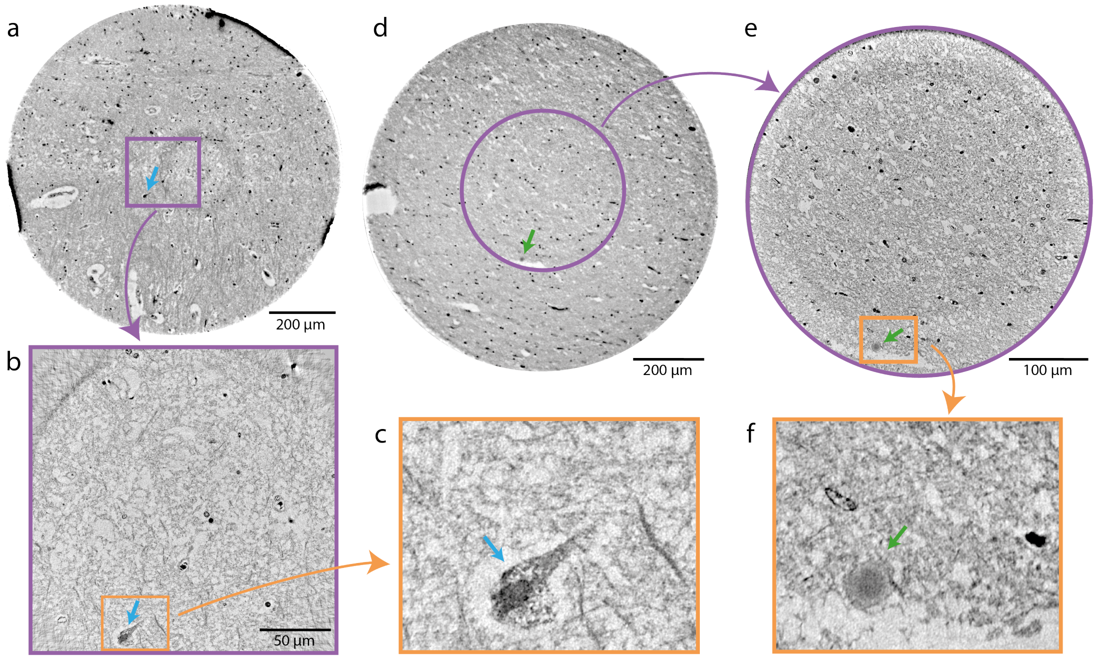

Figure 10: Comparison of data acquired at the parallel beam SR1 setup and the high-resolution SR2 setup. **a** Slice through reconstructed electron density of a 1 mm brain punch from patient with granulovacuolar degeneration (blue arrow) acquired at SR1 setup. **b** Same region as in **a**, recorded with high resolution SR2 setup with (**c**) zoom at granulovacuolar degeneration. **d** Slice through reconstructed electron density of a 1 mm brain punch from patient with Lewy bodies (green arrow) acquired at SR1 setup. **e** Same region as in **d**, recorded with high resolution SR2 setup with (**f**) zoom at a Lewy body.

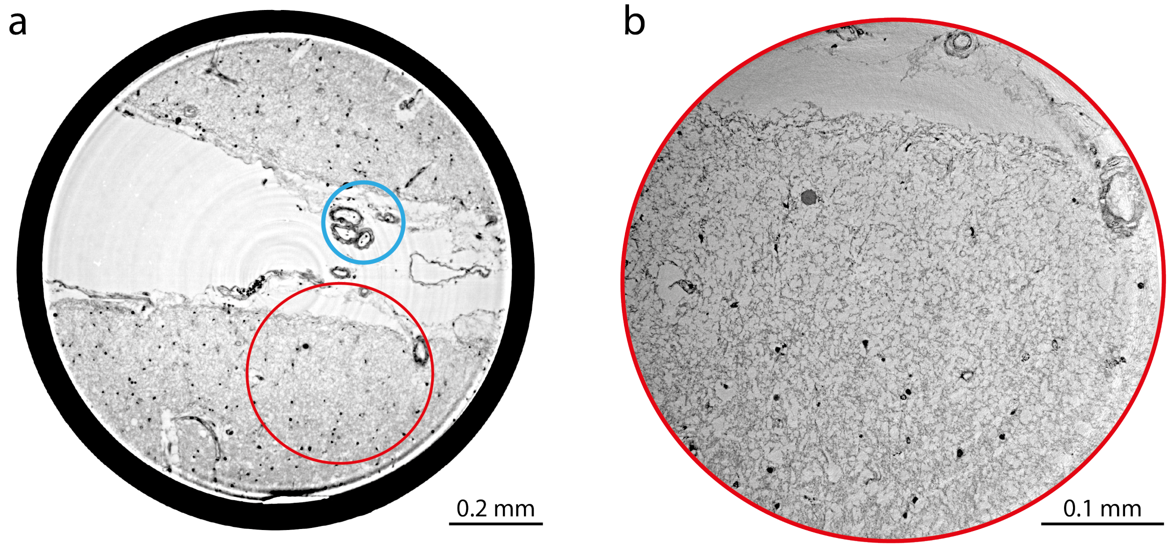

Figure 11: Vascular amyloid deposition in cerebral amyloid angiopathy (CAA). **a** Virtual section covering the entire 1 mm diameter of the punch biopsy (SR1-setup). **b** Section through a subset of the volume covered by the high resolution scan (SR2-setup).

Supporting Figures: Additional Visualization of Hirano bodies

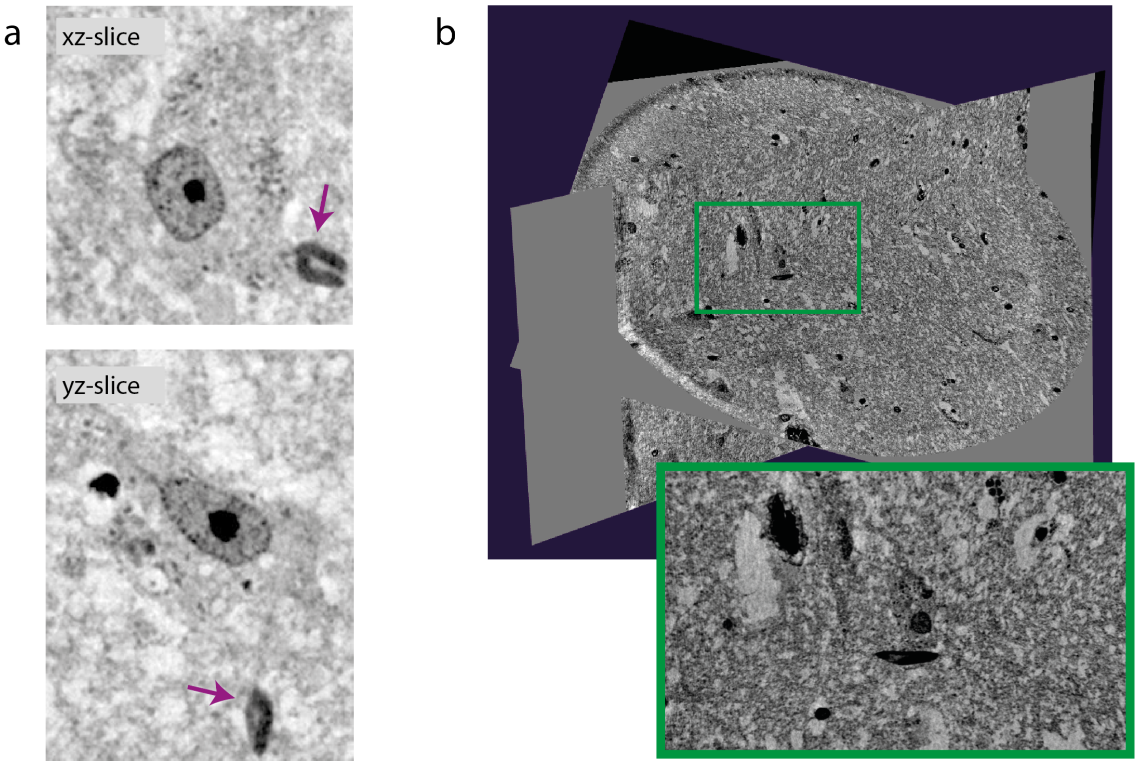

Figure 12: **a** Orthogonal sections through the same Hirano body as shown in the main manuscript. **b** Differently oriented slices through three dimensional volume (visualization with viewer by Histomography GmbH).

Supporting Figures: Hirano bodies and Neurofibrillary Tangles with Correlative Immunochemistry

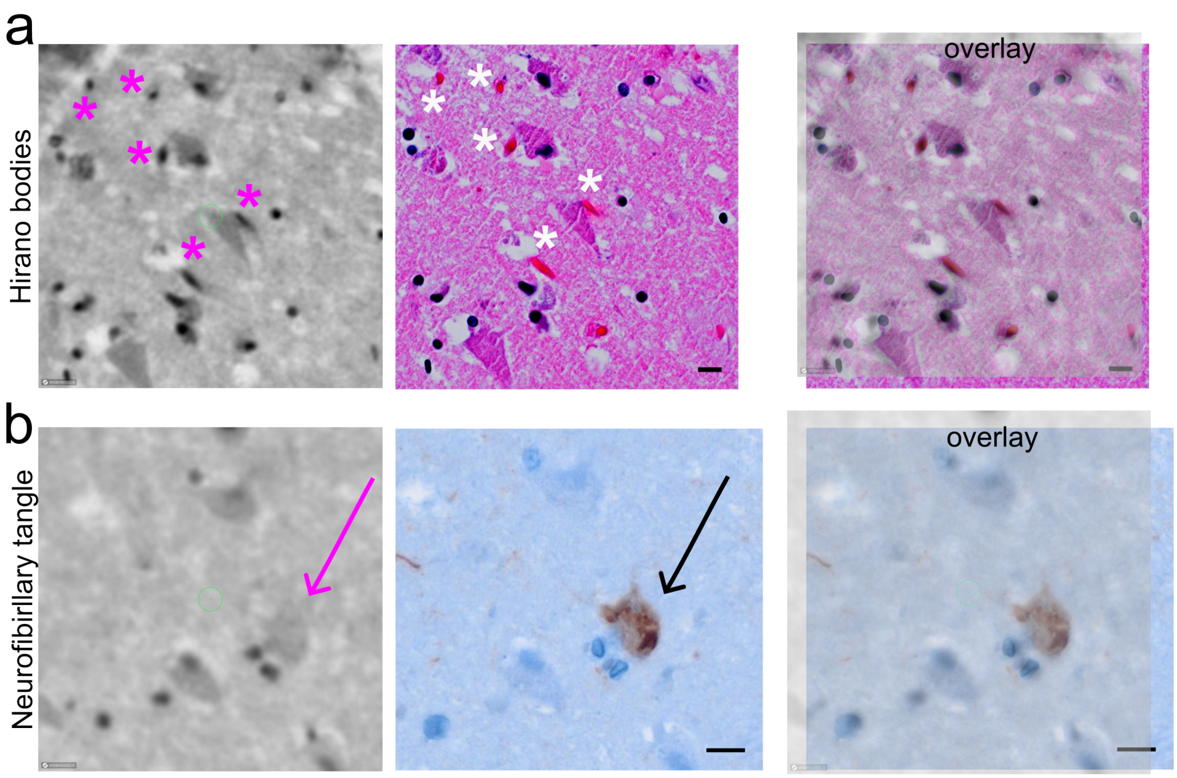

Figure 13: Direct superposition of histology/immunohistochemistry and XPCT. **a** Several Hirano bodies (*) in case 1 stained by HE after XPCT and corresponding SR1-recording manually overlayed by 3D plane after adjustment of height and tilting. **b** Single neurofibrillary tangle (arrow) stained by at8 antibody overlayed with SR1-recording. Scale bar 10 µm.

Supporting Figures: Neurofibrillary Tangles Load across Cohort

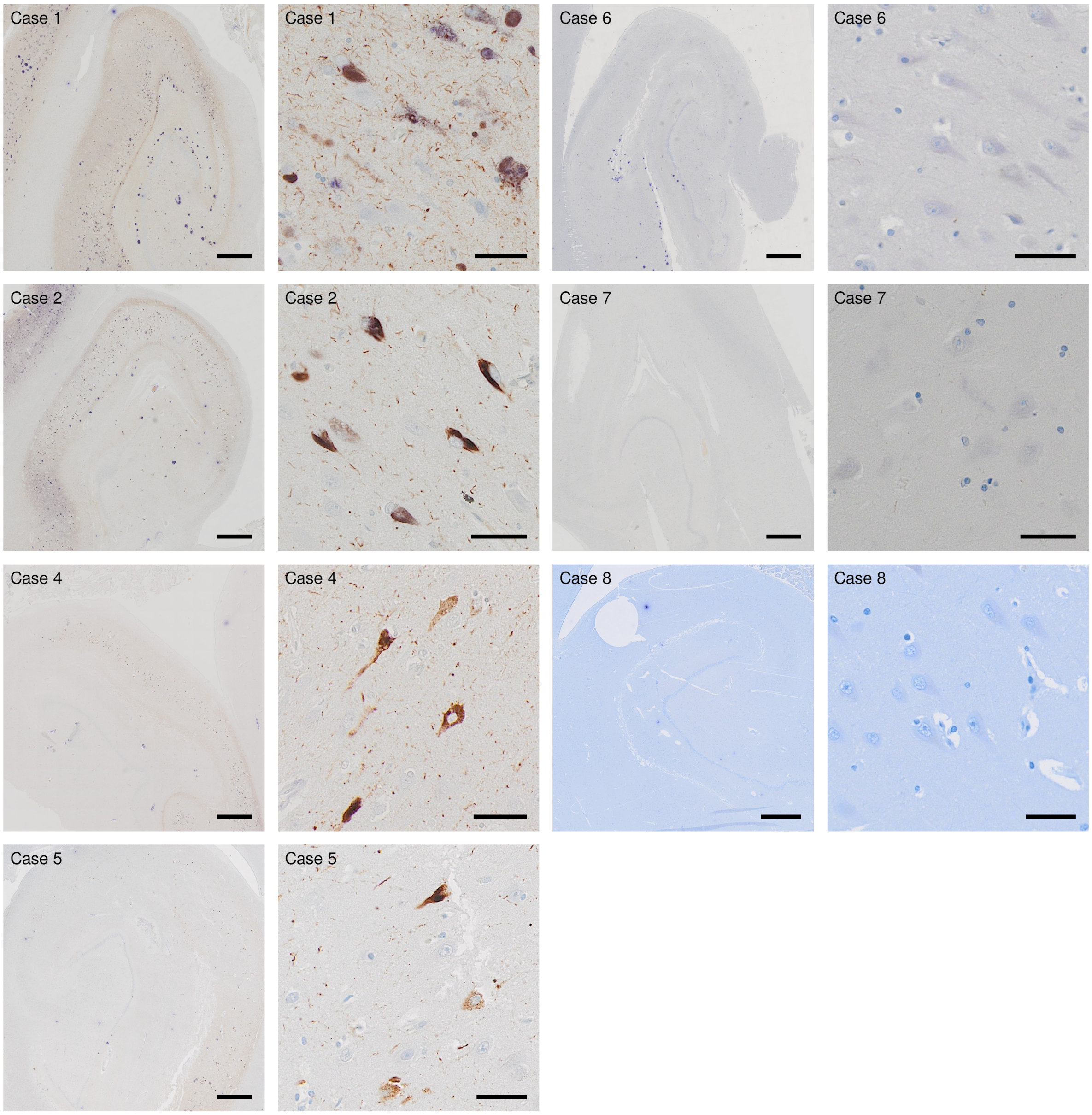

Figure 14: All cases which underwent segmentation and electron density quantification of CA1 neurons stained in a hippocampal section for hyperphosphorylated tau protein (at8 antibody). Scale bar (column wise 1 mm, 50 µm, 1 mm, 50 µm).

References

Berg, S., Kutra, D., Kroeger, T., Straehle, C. N., Kausler, B. X., Haubold, C., Schiegg, M., Ales, J., Beier, T., Rudy, M., Eren, K., Cervantes, J. I., Xu, B., Beuttenmueller, F., Wolny, A., Zhang, C., Koethe, U., Hamprecht, F. A. & Kreshuk, A. (2019), 'ilastik: interactive machine learning for (bio)image analysis', *Nature Methods* **16**(12), 1226–1232. Number: 12 Publisher: Nature Publishing Group.
URL: *https://www.nature.com/articles/s41592-019-0582-9*

Boergens, K. M., Berning, M., Bocklisch, T., Bräunlein, D., Drawitsch, F., Frohnhofen, J., Herold, T., Otto, P., Rzepka, N., Werkmeister, T., Werner, D., Wiese, G., Wissler, H. & Helmstaedter, M. (2017), 'webKnossos: efficient online 3D data annotation for connectomics', *Nature Methods* **14**(7), 691–694. Publisher: Nature Publishing Group.
URL: *https://www.nature.com/articles/nmeth.4331*

Cloetens, P., Ludwig, W., Baruchel, J., Van Dyck, D., Van Landuyt, J., Guigay, J. P. & Schlenker, M. (1999), 'Holotomography: Quantitative phase tomography with micrometer resolution using hard synchrotron radiation x rays', *Applied Physics Letters* **75**(19), 2912–2914.
URL: *http://aip.scitation.org/doi/10.1063/1.125225*

Henke, B. L., Gullikson, E. M. & Davis, J. C. (1993), 'X-Ray Interactions: Photoabsorption, Scattering, Transmission, and Reflection at E = 50-30,000 eV, Z = 1-92', *Atomic Data and Nuclear Data Tables* **54**(2), 181–342.
URL: *https://www.sciencedirect.com/science/article/pii/S0092640X83710132*

Kaye, G. W. C. & Laby, T. H. (1995), *Tables of physical and chemical constants*, 16th ed edn, Longman Scientific & Technical, Essex, England ; New York.

Lohse, L., Robisch, A.-L., Töpperwien, M., Maretzke, S., Krenkel, M., Hagemann, J. & Salditt, T. (2020), 'A phase-retrieval toolbox for X-ray holography and tomography', *Journal of Synchrotron Radiation* **27**.

Münch, B., Trtik, P., Marone, F. & Stampanoni, M. (2009), 'Stripe and ring artifact removal with combined wavelet—Fourier filtering', *Optics Express* **17**(10), 8567.
URL: *https://www.osapublishing.org/oe/abstract.cfm?uri=oe-17-10-8567*

Reichmann, J., Verleden, S. E., Kühnel, M., Kamp, J. C., Werlein, C., Neubert, L., Müller, J.-H., Bui, T. Q., Ackermann, M., Jonigk, D. & Salditt, T. (2023), 'Human lung virtual histology by multi-scale x-ray phase-contrast computed tomography', *Physics in Medicine & Biology* **68**(11), 115014. Publisher: IOP Publishing.
URL: *https://dx.doi.org/10.1088/1361-6560/acd48d*

Robisch, A.-L., Eckermann, M., Töpperwien, M., Meer, F. v. d., Stadelmann-Nessler, C. & Salditt, T. (2020), 'Nanoscale x-ray holotomography of human brain tissue with phase retrieval based on multienergy recordings', *Journal of Medical Imaging* **7**(1), 013501. Publisher: SPIE.
URL: *https://www.spiedigitallibrary.org/journals/journal-of-medical-*

imaging/volume-7/issue-1/013501/Nanoscale-x-ray-holotomography-of-human-brain-tissue-with-phase/10.1117/1.JMI.7.1.013501.full

van Aarle, W., Palenstijn, W. J., De Beenhouwer, J., Altantzis, T., Bals, S., Batenburg, K. J. & Sijbers, J. (2015), 'The ASTRA Toolbox: A platform for advanced algorithm development in electron tomography', *Ultramicroscopy* **157**, 35–47.
URL: *https://www.sciencedirect.com/science/article/pii/S0304399115001060*

van Heel, M. & Schatz, M. (2005), 'Fourier shell correlation threshold criteria', *Journal of Structural Biology* **151**(3), 250–262.
URL: *https://linkinghub.elsevier.com/retrieve/pii/S1047847705001292*

Yu, B., Weber, L., Pacureanu, A., Langer, M., Olivier, C., Cloetens, P. & Peyrin, F. (2018), 'Evaluation of phase retrieval approaches in magnified X-ray phase nano computerized tomography applied to bone tissue', *Optics Express* **26**(9), 11110–11124. Publisher: Optica Publishing Group.
URL: *https://opg.optica.org/oe/abstract.cfm?uri=oe-26-9-11110*
